# Supplementary material for: Synthesis of New Proteomimetic Quinazolinone Alkaloids and Evaluation of Their Neuroprotective and Antitumor Effects
Source: Molecules. 2019 Feb 1;24(3):534. doi: 10.3390/molecules24030534 (PMC6384550; doi:10.3390/molecules24030534)
Supplement: Supplementary file 1 [file molecules-24-00534-s001.pdf]

Supplementary information

Synthesis of new proteomimetic quinazolinone alkaloids and evaluation of their neuroprotective and antitumor effects

Solida Long [1], Diana I. S. P. Resende [1,2], Anake Kijjoa [2,3], Artur M. S. Silva [4], Ricardo Fernandes [5,6], Cristina P. R. Xavier [5,6], M. Helena Vasconcelos [5,6,7], Emília Sousa [1,2,*], and Madalena M. M. Pinto [1,2]

[1] Laboratory of Organic and Pharmaceutical Chemistry (LQOF), Department of Chemical Sciences, Faculty of Pharmacy, University of Porto, Rua de Jorge Viterbo Ferreira, 228, 4050-313 Porto, Portugal
[2] Interdisciplinary Centre of Marine and Environmental Research (CIIMAR), 4450-208 Matosinhos, Portugal
[3] *ICBAS*-Instituto de Ciências Biomédicas Abel Salazar, University of Porto, 4050-313 Porto, Portugal
[4] Organic Chemistry and Natural Products Unit (QOPNA), Department of Chemistry, University of Aveiro, 3810-193 Aveiro, Portugal
[5] Instituto de Investigação e Inovação em Saúde (i3S), University of Porto, 4200-135 Porto, Portugal.
[6] Cancer Drug Resistance Group, Institute of Molecular Pathology and Immunology of the University of Porto (IPATIMUP), 4200-135, Porto, Portugal
[7] Laboratory of Microbiology, Department of Biological Sciences, Faculty of Pharmacy, University of Porto, 4050-313 Porto, Portugal
* Correspondence: esousa@ff.up.pt; Tel.: +351-2-2042-8689

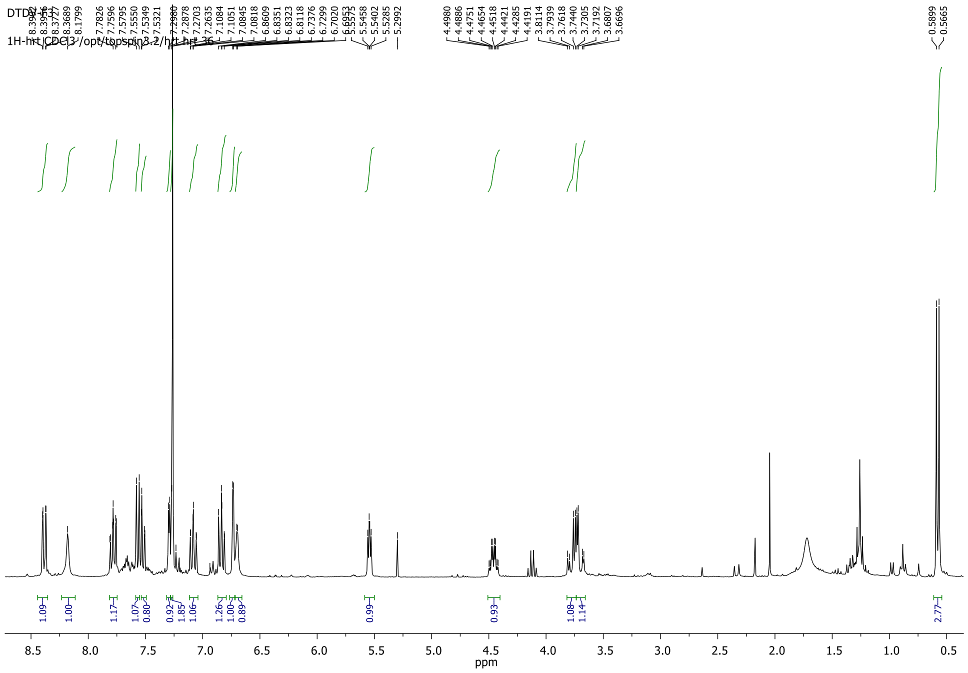

Figure S1. ¹H NMR spectrum of *(1R,4R)-4-((1H-indol-3-yl)methyl)-1-((R)-methyl)-1,2-dihydro-6H-pyrazino[2,1-b]quinazoline-3,6(4H)-dione* (**1**) ($CDCl_3$, 300, MHz).

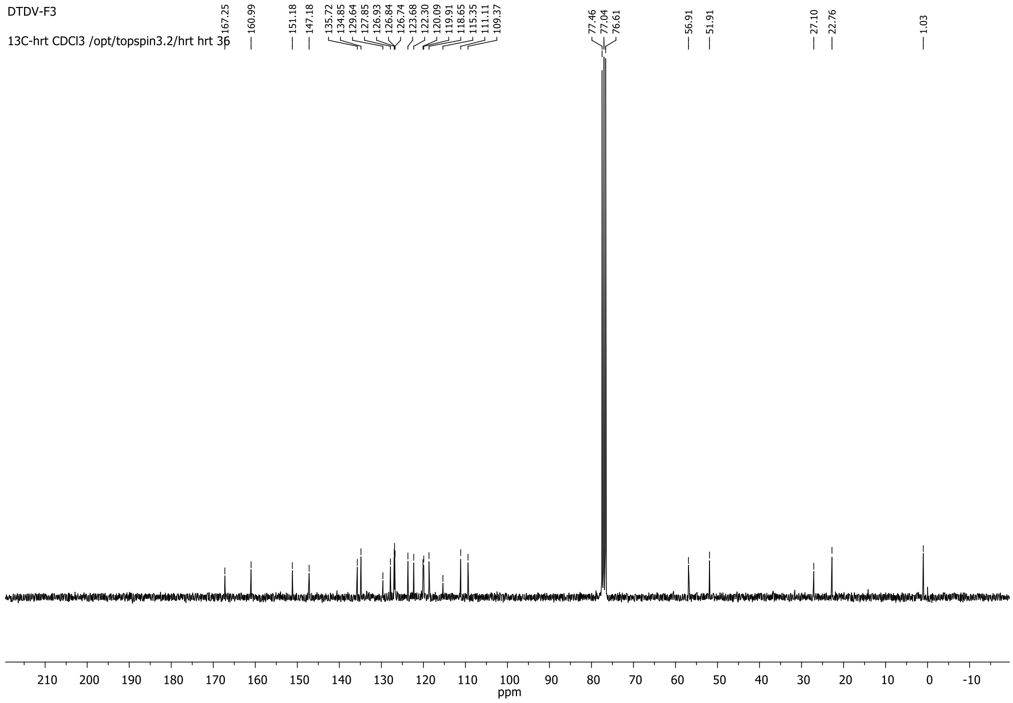

Figure S2. ¹³C NMR spectrum of *(1R,4R)-4-((1H-indol-3-yl)methyl)-1-((R)-methyl)-1,2-dihydro-6H-pyrazino[2,1-b]quinazoline-3,6(4H)-dione* (**1**) ($CDCl_3$, 75, MHz).

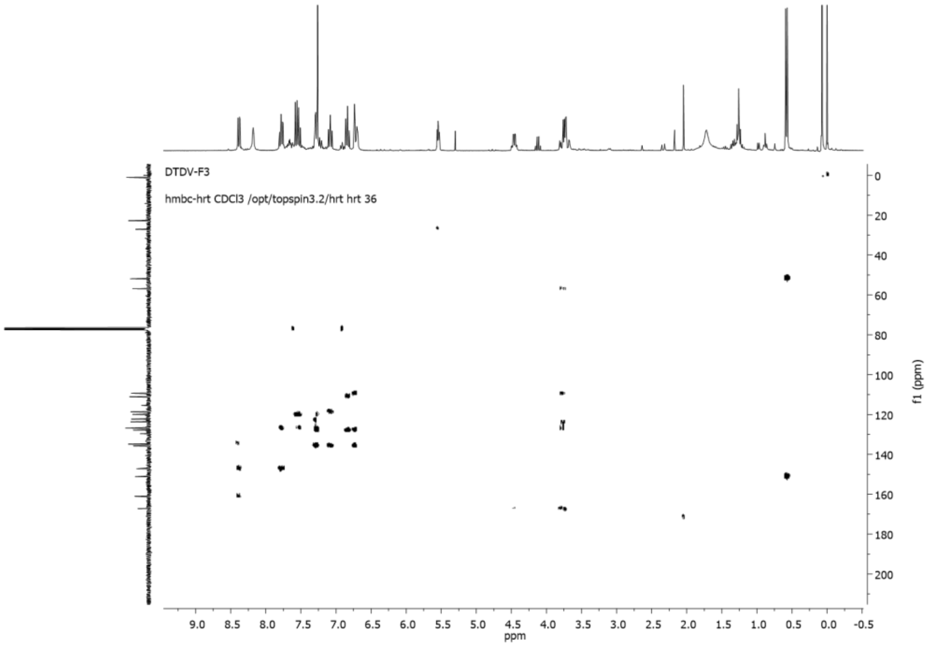

Figure S3. HMBC spectrum of *(1R,4R)-4-((1H-indol-3-yl)methyl)-1-((R)-methyl)-1,2-dihydro-6H-pyrazino[2,1-b]quinazoline-3,6(4H)-dione* (***1***) ($CDCl_3$, 300 MHz).

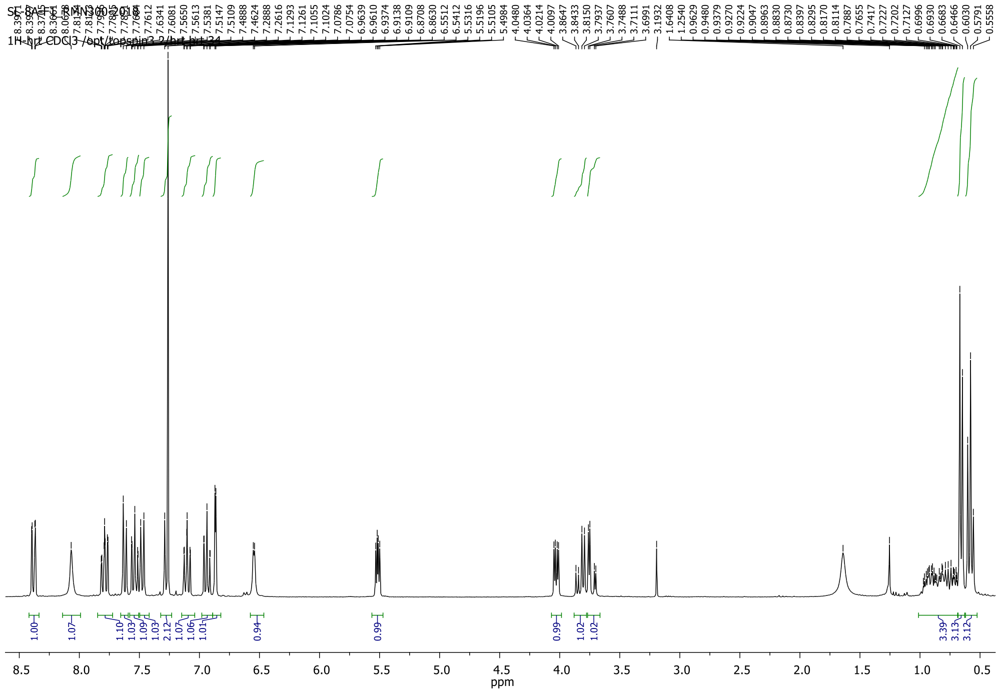

Figure S4. ^{1}H NMR spectrum of *(1S,4S)-4-((1H-indol-3-yl)methyl)-1-((S)-sec-butyl)-1,2-dihydro-6H-pyrazino[2,1-b]quinazoline-3,6(4H)-dione* (**2**) ($CDCl_3$, 300, MHz).

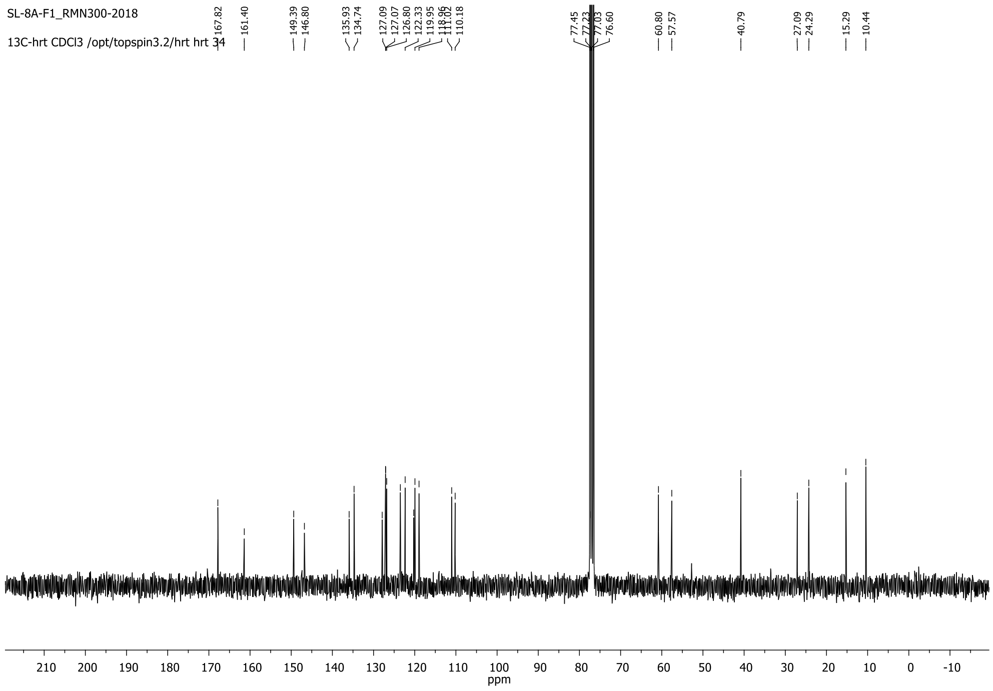

Figure S5. ^{13}C NMR spectrum of *(1S,4S)-4-((1H-indol-3-yl)methyl)-1-((S)-sec-butyl)-1,2-dihydro-6H-pyrazino[2,1-b]quinazoline-3,6(4H)-dione* (**2**) (CDCl3, 75, MHz).

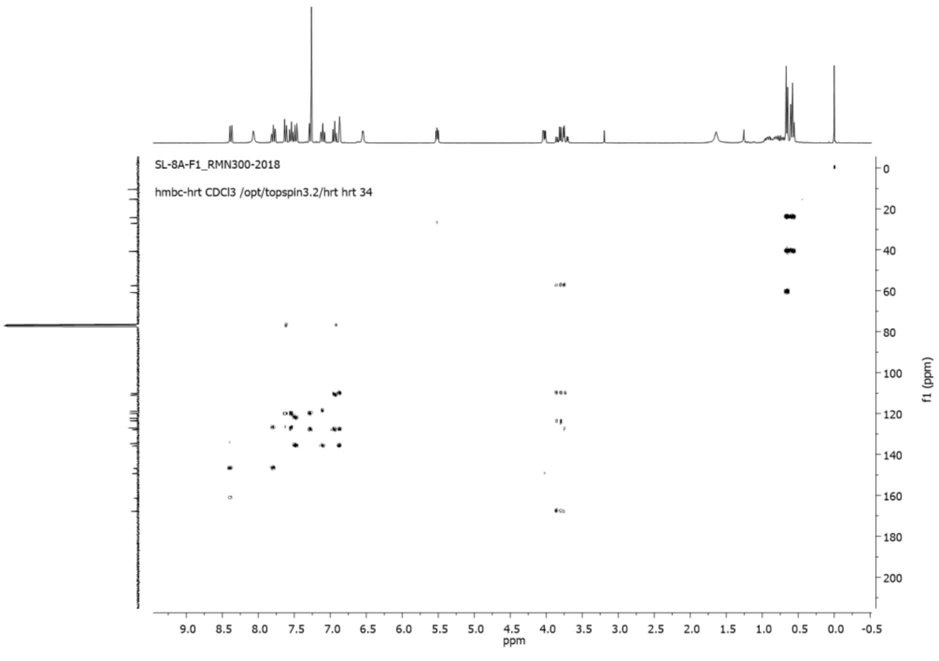

Figure S6. HMBC spectrum of *(1S,4S)-4-((1H-indol-3-yl)methyl)-1-((S)-sec-butyl)-1,2-dihydro-6H-pyrazino[2,1-b]quinazoline-3,6(4H)-dione* (**2**) (CDCl3, 300 MHz).

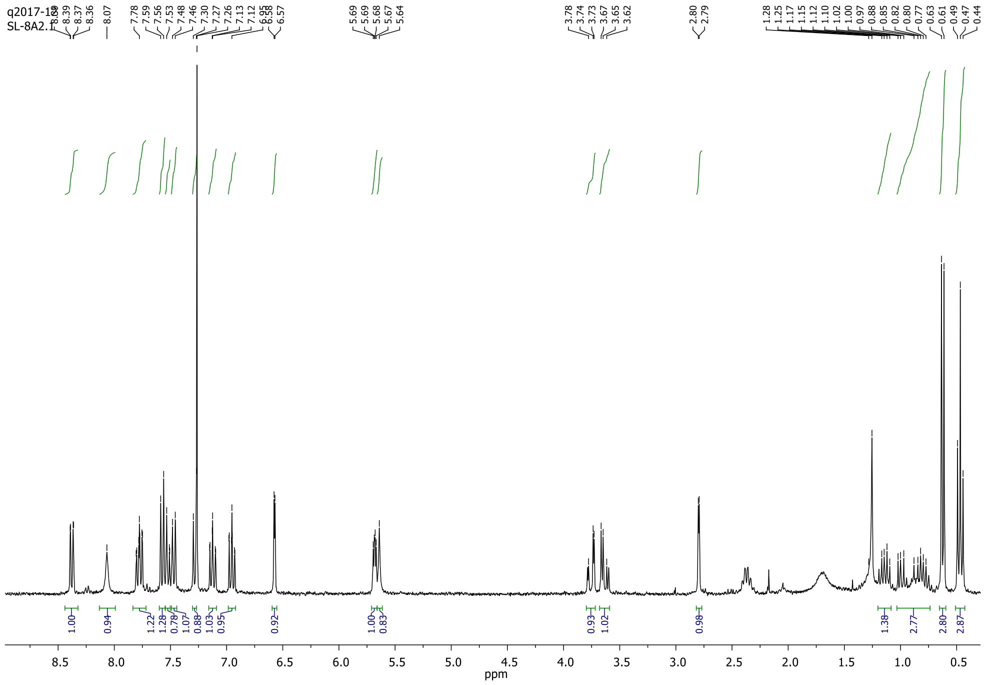

Figure S7. ^{1}H NMR spectrum of *(1R,4S)-4-((1H-indol-3-yl)methyl)-1-((S)-sec-butyl)-1,2-dihydro-6H-pyrazino[2,1-b]quinazoline-3,6(4H)-dione* (**4**) ($CDCl_3$, 300, MHz).

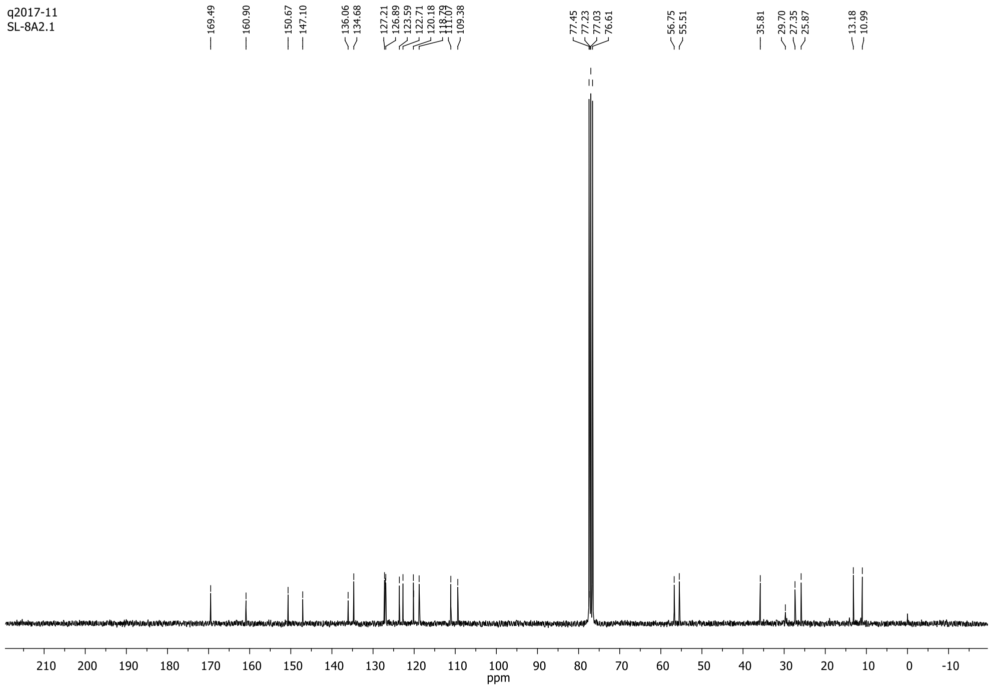

Figure S8. ^{13}C NMR spectrum of *(1R,4S)-4-((1H-indol-3-yl)methyl)-1-((S)-sec-butyl)-1,2-dihydro-6H-pyrazino[2,1-b]quinazoline-3,6(4H)-dione* (**4**) ($CDCl_3$, 75, MHz).

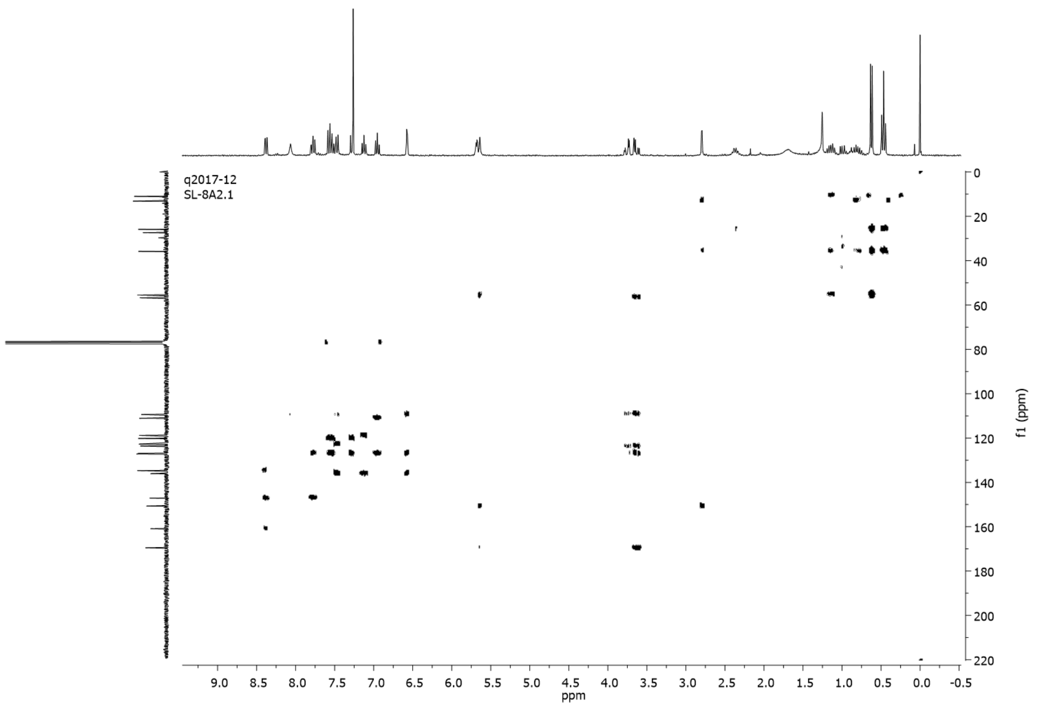

Figure S9. HMBC spectrum of *(1R,4S)-4-((1H-indol-3-yl)methyl)-1-((S)-sec-butyl)-1,2-dihydro-6H-pyrazino[2,1-b]quinazoline-3,6(4H)-dione* (**4**) ($CDCl_3$, 300, MHz).

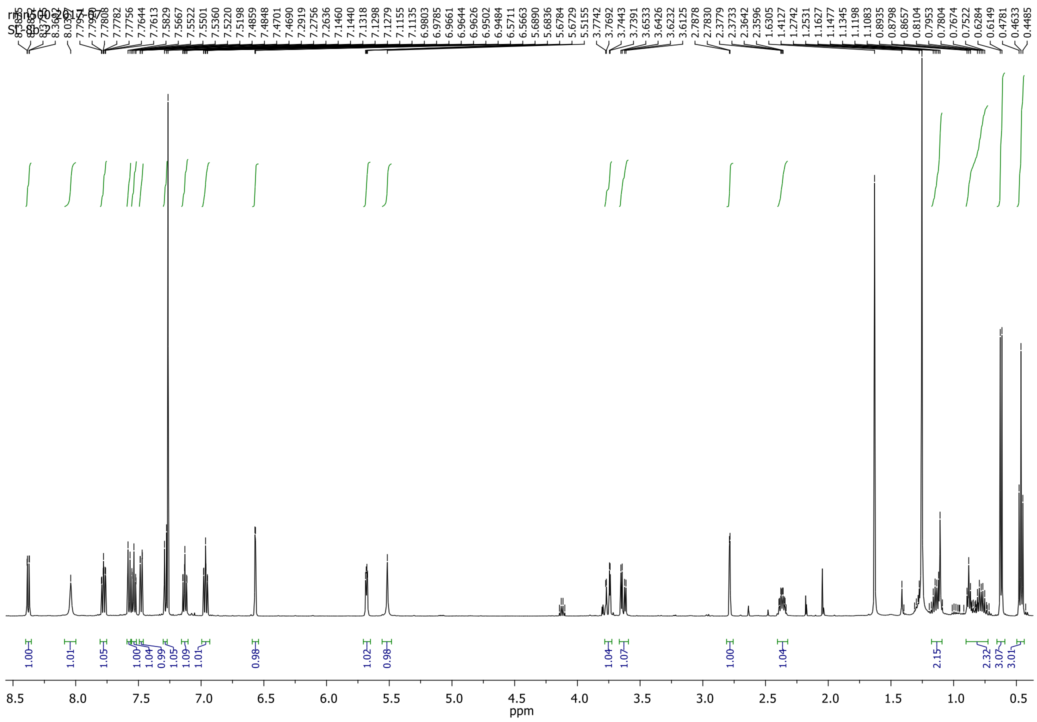

Figure S10. ^{1}H NMR spectrum of *(1S,4R)-4-((1H-indol-3-yl)methyl)-1-((S)-sec-butyl)-1,2-dihydro-6H-pyrazino[2,1-b]quinazoline-3,6(4H)-dione* (**5**) ($CDCl_3$, 300, MHz).

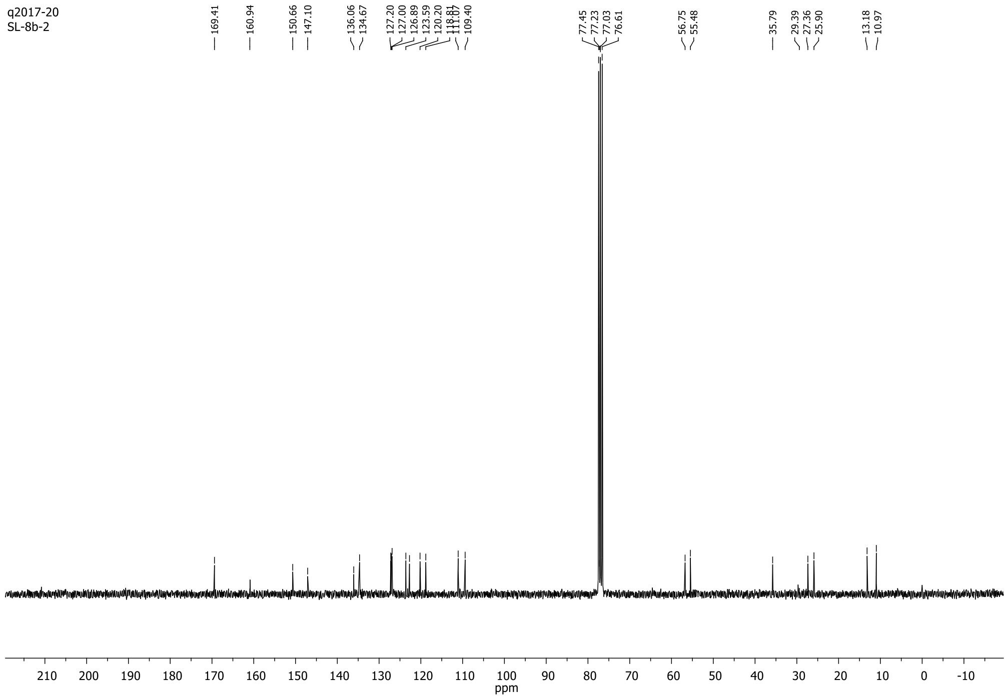

Figure S11. ^{13}C NMR spectrum of *(1S,4R)-4-((1H-indol-3-yl)methyl)-1-((S)-sec-butyl)-1,2-dihydro-6H-pyrazino[2,1-b]quinazoline-3,6(4H)-dione* (**5**) ($CDCl_3$, 75, MHz).

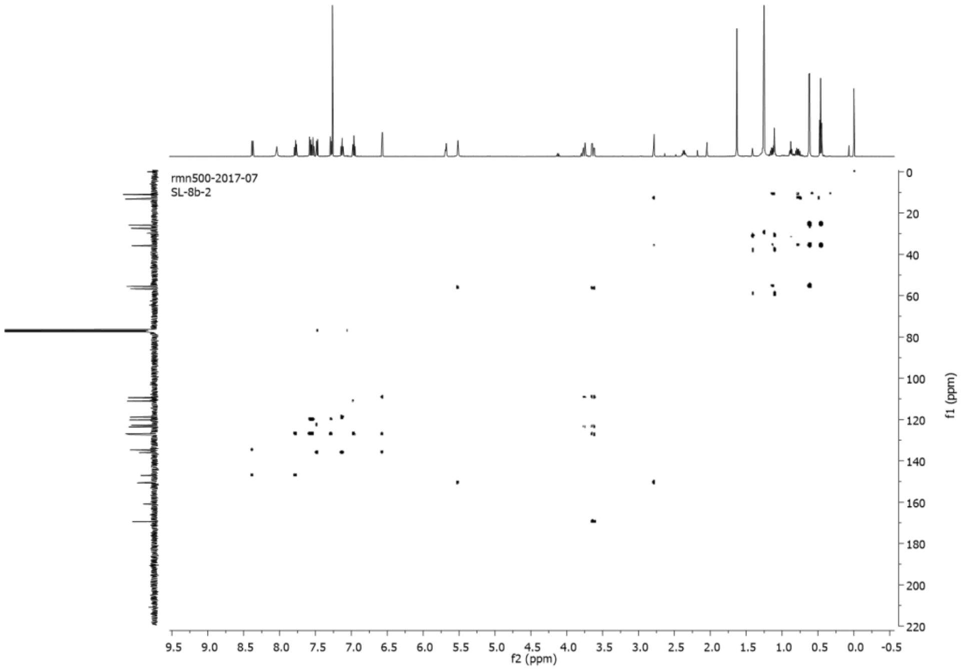

Figure S12. HMBC spectrum of *(1S,4R)-4-((1H-indol-3-yl)methyl)-1-((S)-sec-butyl)-1,2-dihydro-6H-pyrazino[2,1-b]quinazoline-3,6(4H)-dione* (**5**) ($CDCl_3$, 300, MHz).

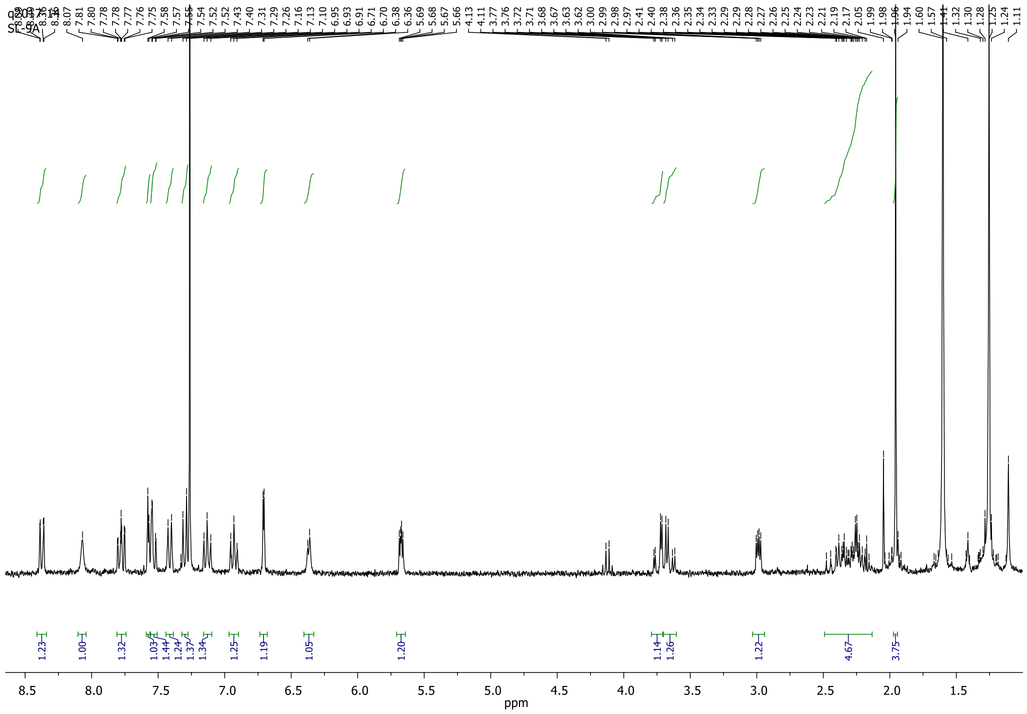

Figure S13: ^{1}H NMR spectrum of *(1R,4S)-4-((1H-indol-3-yl)methyl)-1-(2-(methylthio)ethyl)-1,2-dihydro-6H-pyrazino[2,1-b]quinazoline-3,6(4H)-diones* (**6**) ($CDCl_3$, 300, MHz).

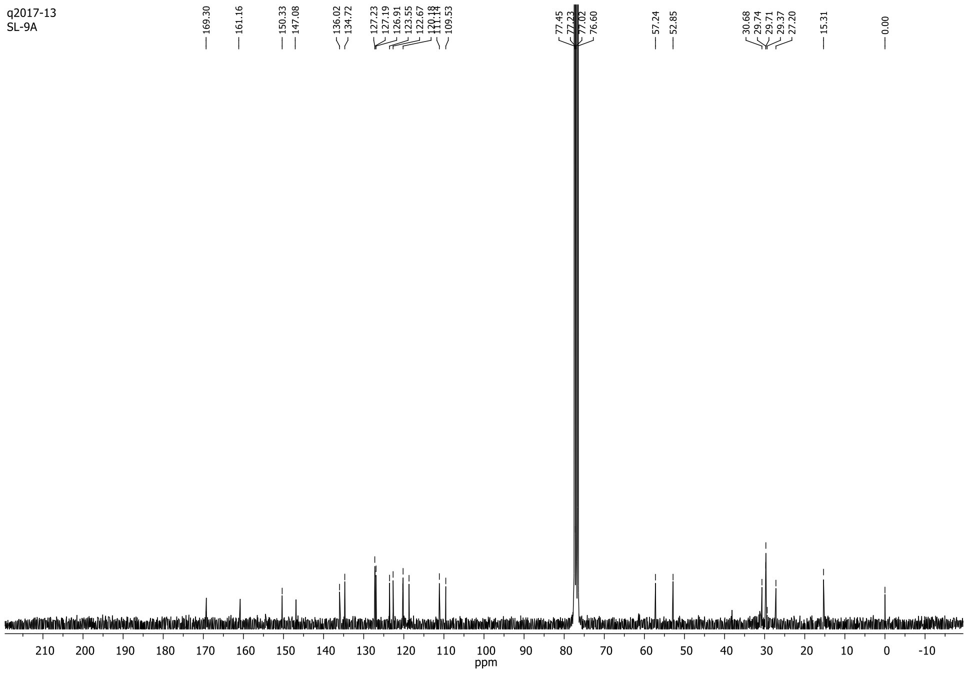

Figure S14: ^{13}C NMR spectrum of *(1R,4S)-4-((1H-indol-3-yl)methyl)-1-(2-(methylthio)ethyl)-1,2-dihydro-6H-pyrazino[2,1-b]quinazoline-3,6(4H)-diones* (**6**) ($CDCl_3$, 75, MHz).

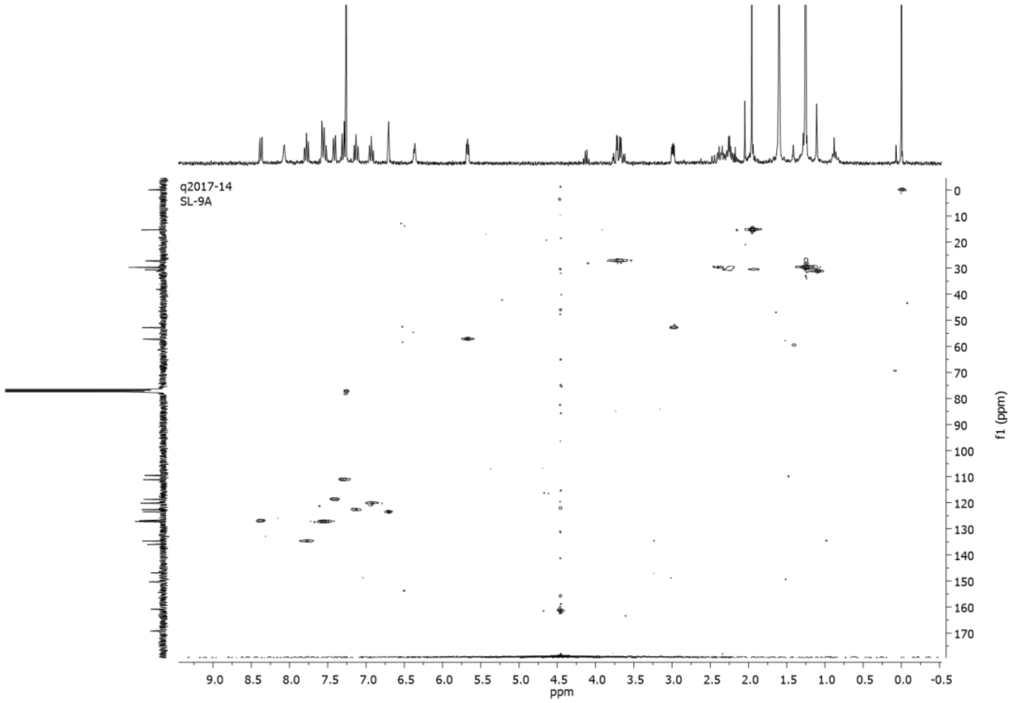

Figure S15: HMBC spectrum of *(1R,4S)-4-((1H-indol-3-yl)methyl)-1-(2-(methylthio)ethyl)-1,2-dihydro-6H-pyrazino[2,1-b]quinazoline-3,6(4H)-diones* (**6**) ($CDCl_3$, 300, MHz).

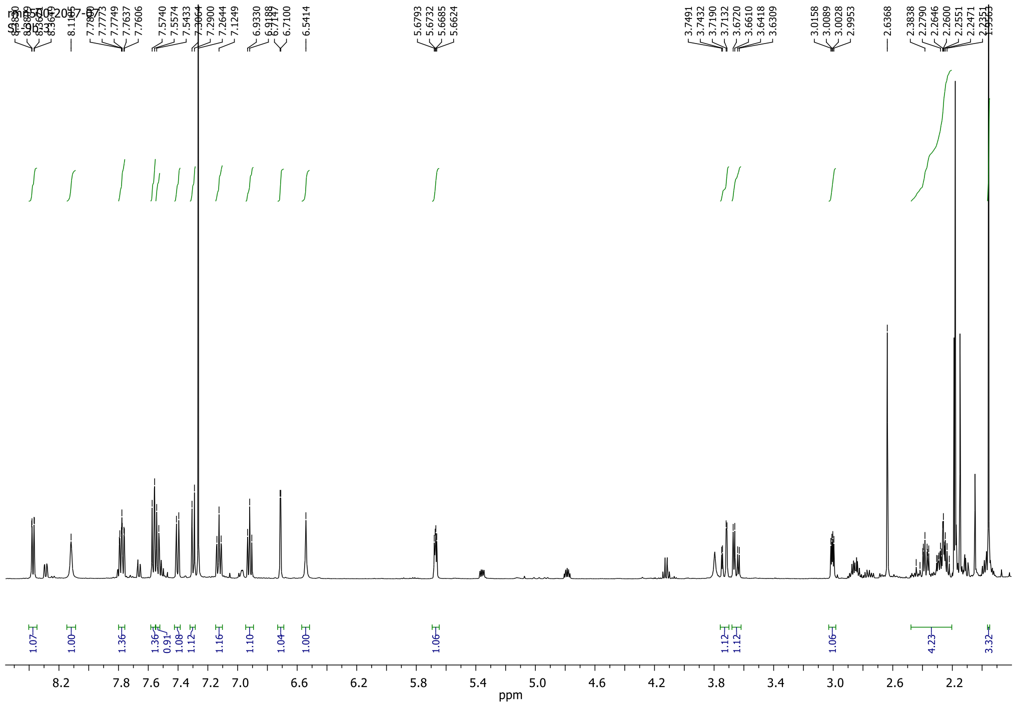

Figure S16: ^{1}H NMR spectrum of *(1S,4R)-4-((1H-indol-3-yl)methyl)-1-(2-(methylthio)ethyl)-1,2-dihydro-6H-pyrazino[2,1-b]quinazoline-3,6(4H)-diones (***7***)* ($CDCl_3$, 300, MHz).

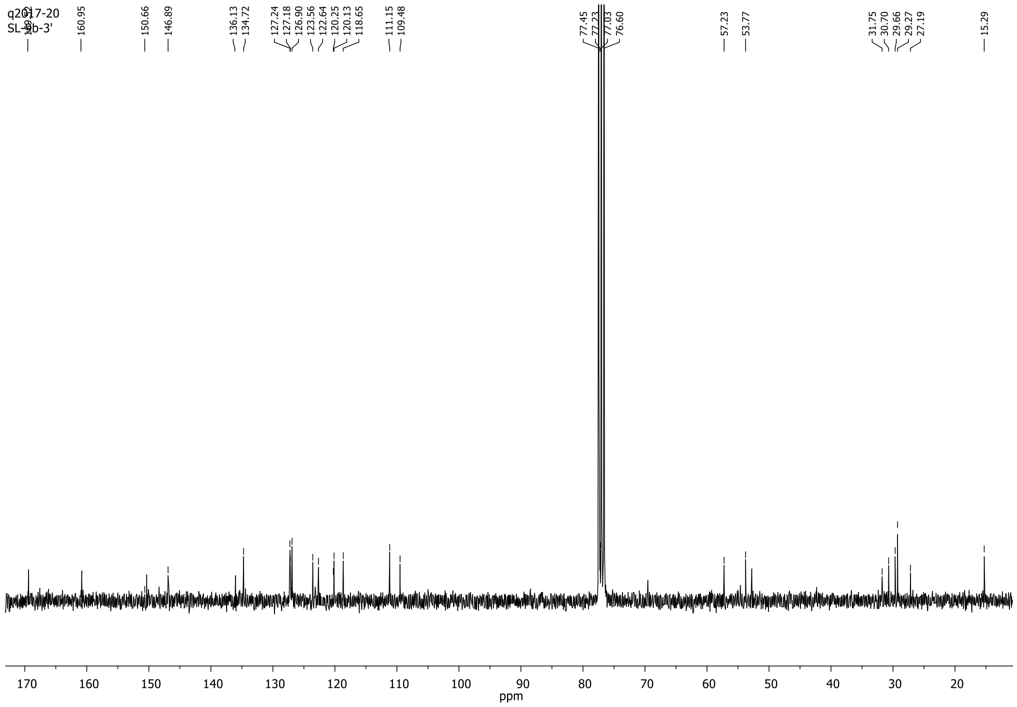

Figure S17: ^{13}C NMR spectrum of *(1S,4R)-4-((1H-indol-3-yl)methyl)-1-(2-(methylthio)ethyl)-1,2-dihydro-6H-pyrazino[2,1-b]quinazoline-3,6(4H)-diones* (**7**) ($CDCl_3$, 75, MHz).

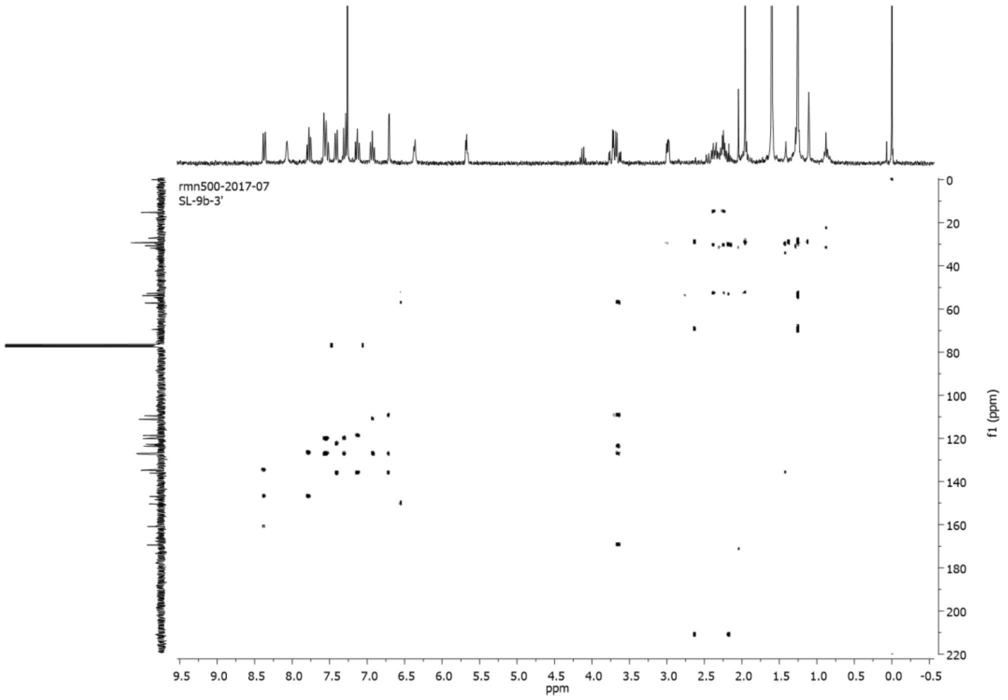

Figure S18: HMBC spectrum of *(1S,4R)-4-((1H-indol-3-yl)methyl)-1-(2-(methylthio)ethyl)-1,2-dihydro-6H-pyrazino[2,1-b]quinazoline-3,6(4H)-diones* (**7**) ($CDCl_3$, 300, MHz).

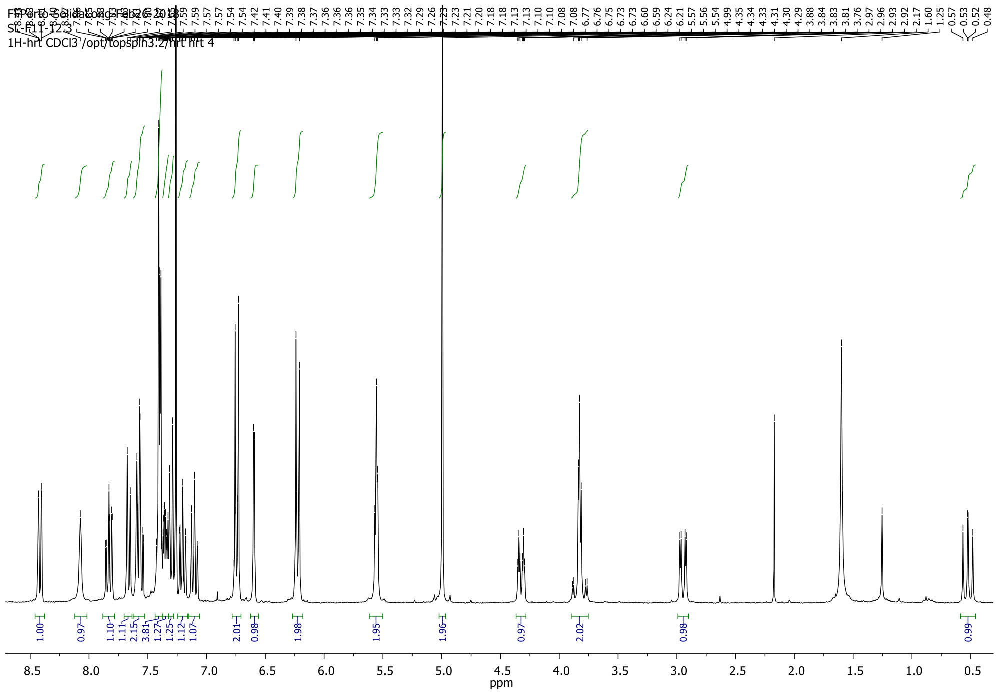

Figure S19: ^{1}H NMR spectrum of *(1S,4S)-4-((1H-indol-3-yl)methyl)-1-(4-(benzyloxy)benzyl)-1,2-dihydro-6H-pyrazino[2,1-b]quinazoline-3,6(4H)-diones* (**8**) (CDCl$_3$, 300, MHz).

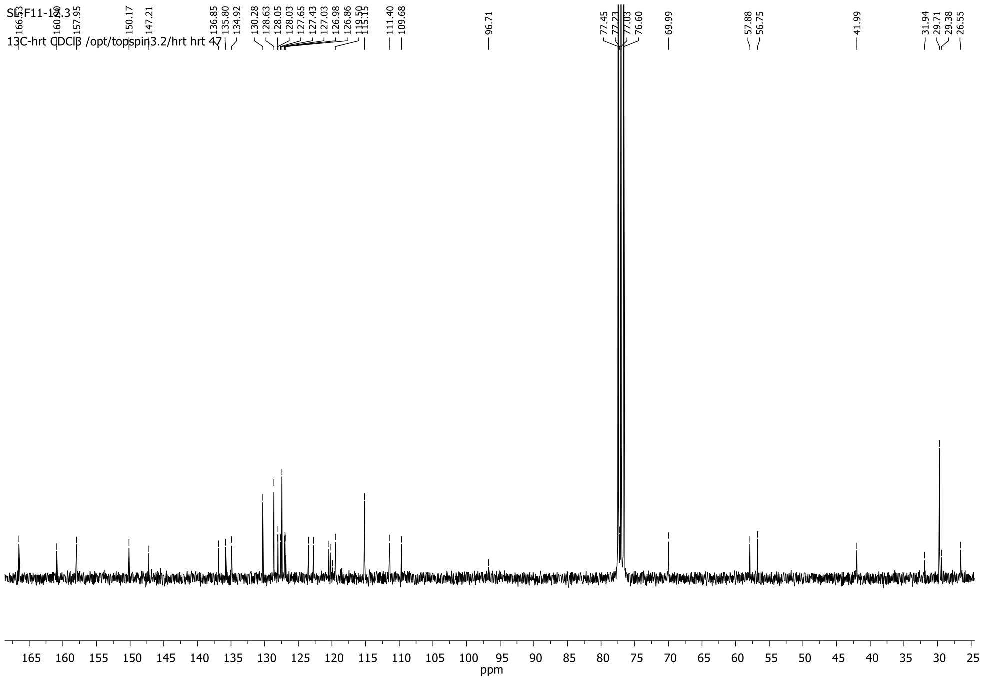

Figure S20: ^{13}C NMR spectrum of *(1S,4S)-4-((1H-indol-3-yl)methyl)-1-(4-(benzyloxy)benzyl)-1,2-dihydro-6H-pyrazino[2,1-b]quinazoline-3,6(4H)-diones* (**8**) (CDCl$_3$, 75, MHz).

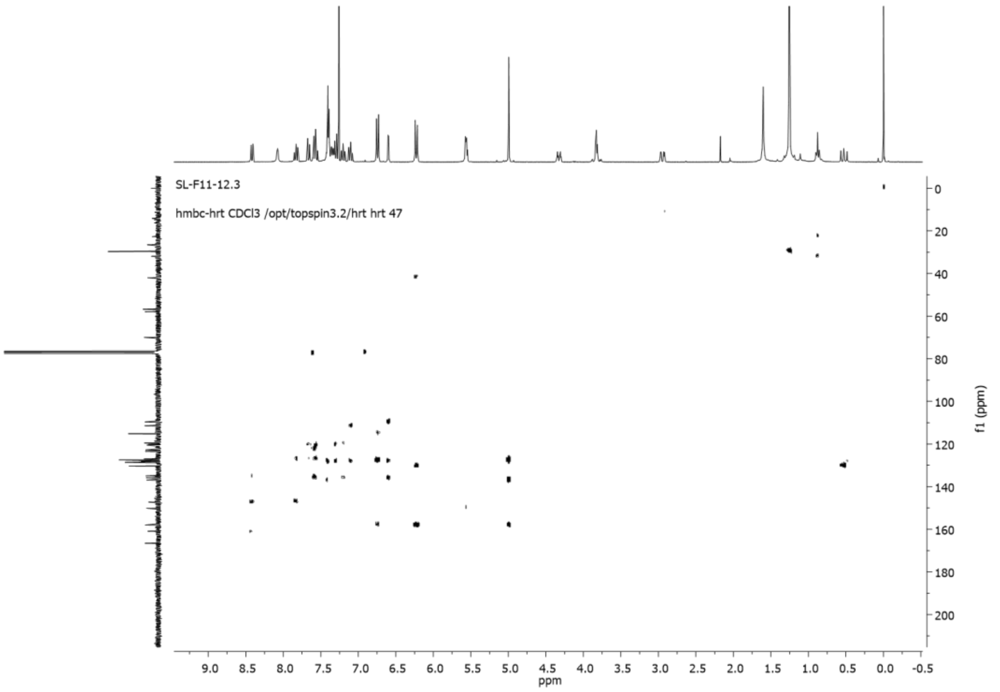

Figure S21: HRMS spectrum of *(1S,4S)-4-((1H-indol-3-yl)methyl)-1-(4-(benzyloxy)benzyl)-1,2-dihydro-6H-pyrazino[2,1-b]quinazoline-3,6(4H)-diones* (**8**) ($CDCl_3$, 300, MHz).

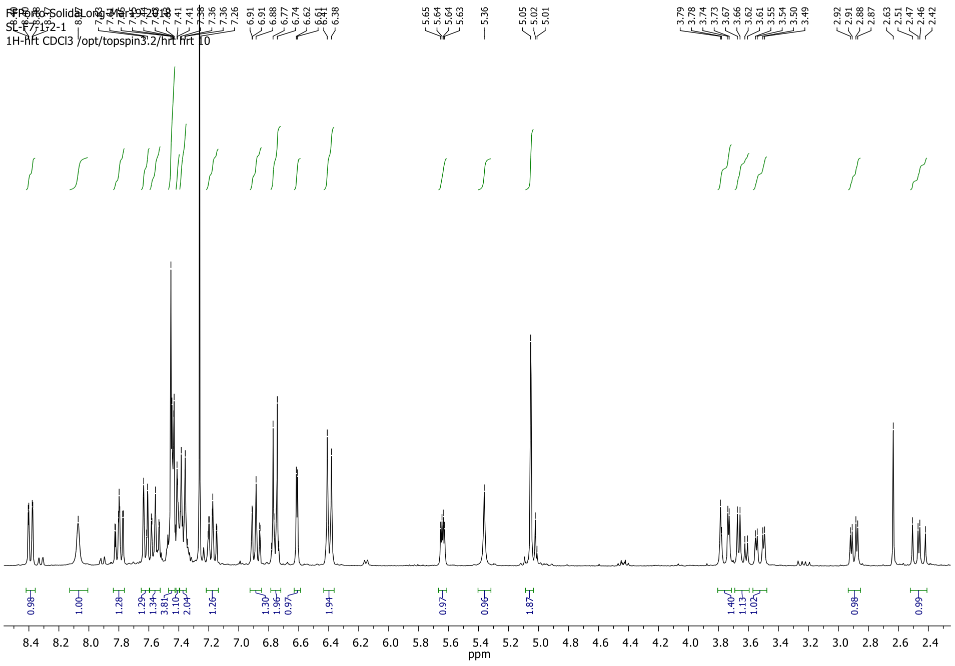

Figure S22: ^{1}H NMR spectrum of *(1R,4S)-4-((1H-indol-3-yl)methyl)-1-(4-(benzyloxy)benzyl)-1,2-dihydro-6H-pyrazino[2,1-b]quinazoline-3,6(4H)-diones* (**9**) ($CDCl_3$, 300, MHz).

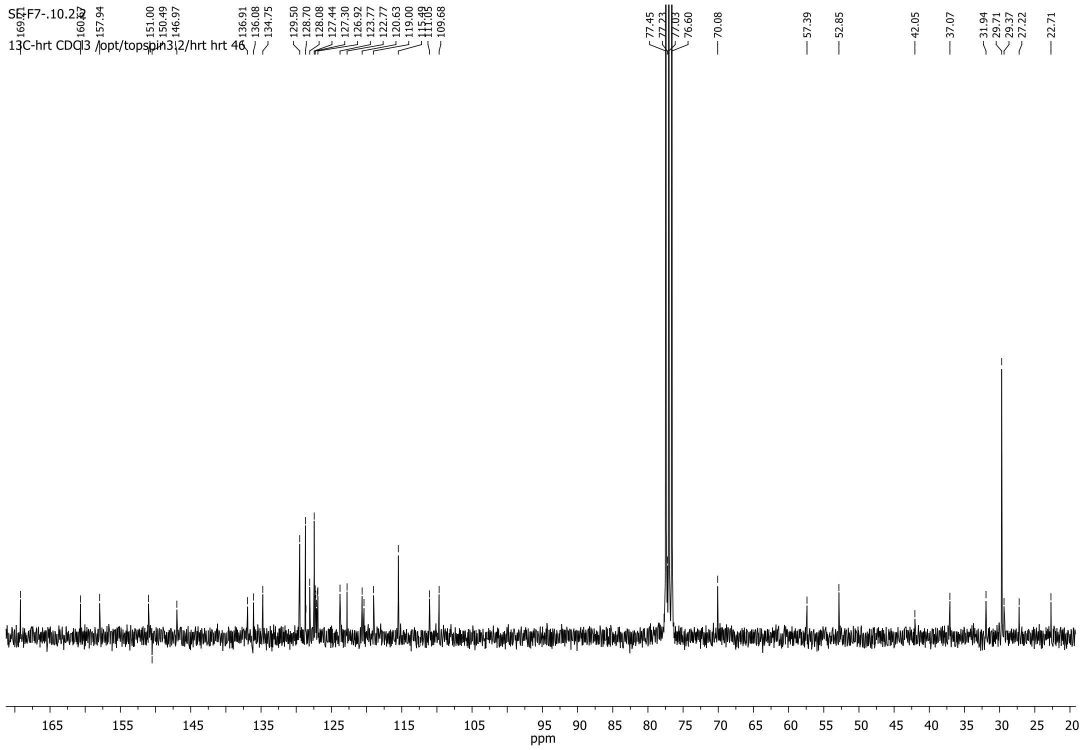

Figure S23: ^{13}C NMR spectrum of *(1R,4S)-4-((1H-indol-3-yl)methyl)-1-(4-(benzyloxy)benzyl)-1,2-dihydro-6H-pyrazino[2,1-b]quinazoline-3,6(4H)-diones (***9***)* (CDCl$_3$, 75, MHz).

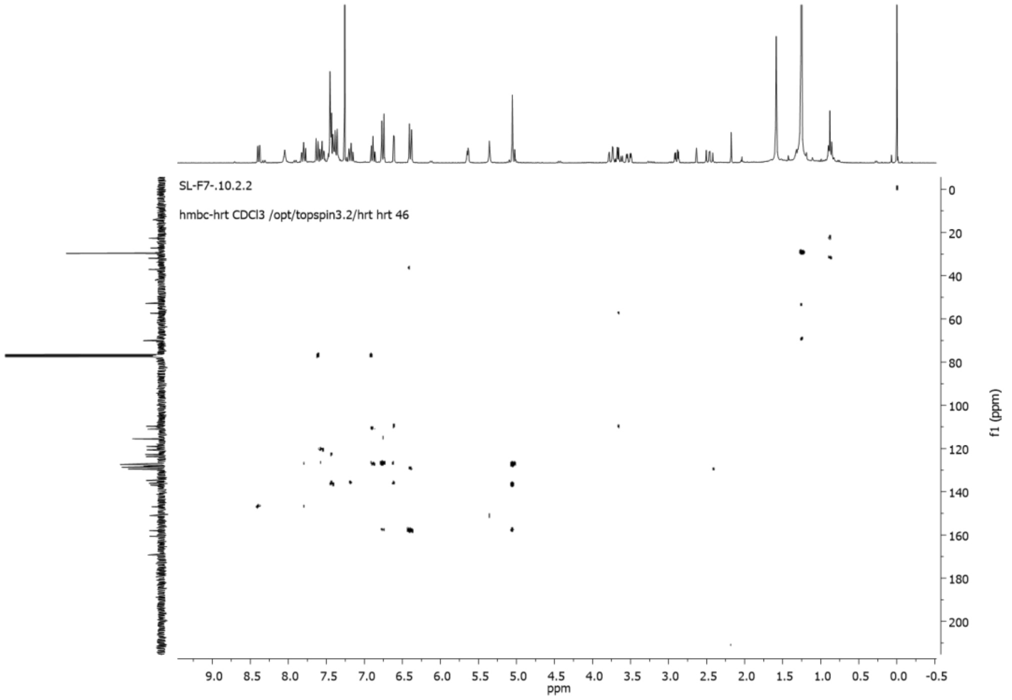

Figure S24: ^{13}C NMR spectrum of *(1R,4S)-4-((1H-indol-3-yl)methyl)-1-(4-(benzyloxy)benzyl)-1,2-dihydro-6H-pyrazino[2,1-b]quinazoline-3,6(4H)-diones (***9***)* (CDCl$_3$, 300, MHz).

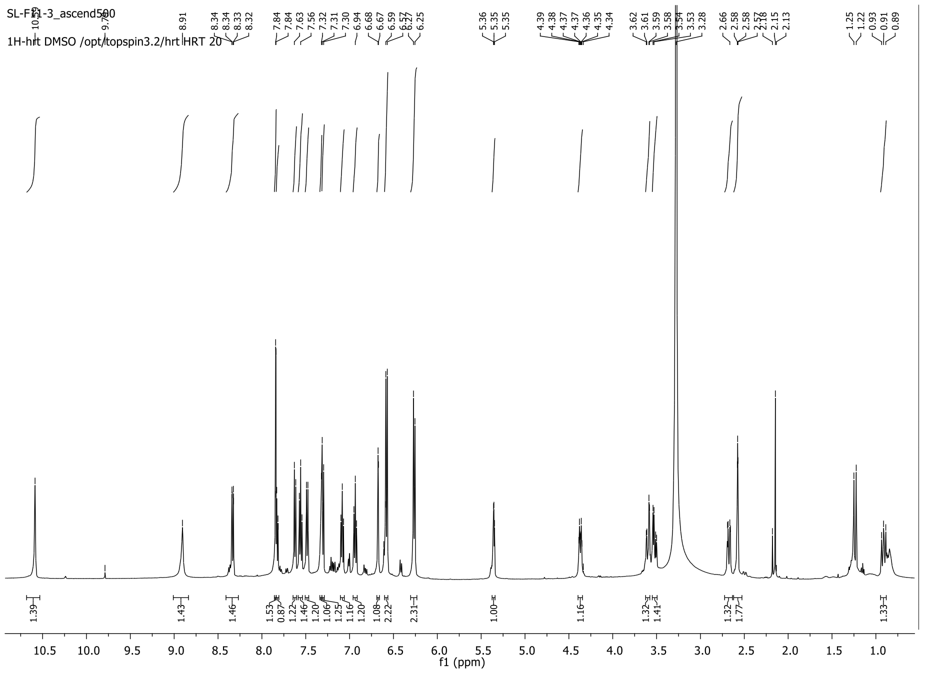

Figure S25: ^{1}H NMR spectrum of *(1S,4S)-4-((1H-indol-3-yl)methyl)-1-(4-hydroxybenzyl)-1,2-dihydro-6H-pyrazino[2,1-b]quinazoline-3,6(4H)-dione* **10** ($CDCl_3$, 300, MHz).

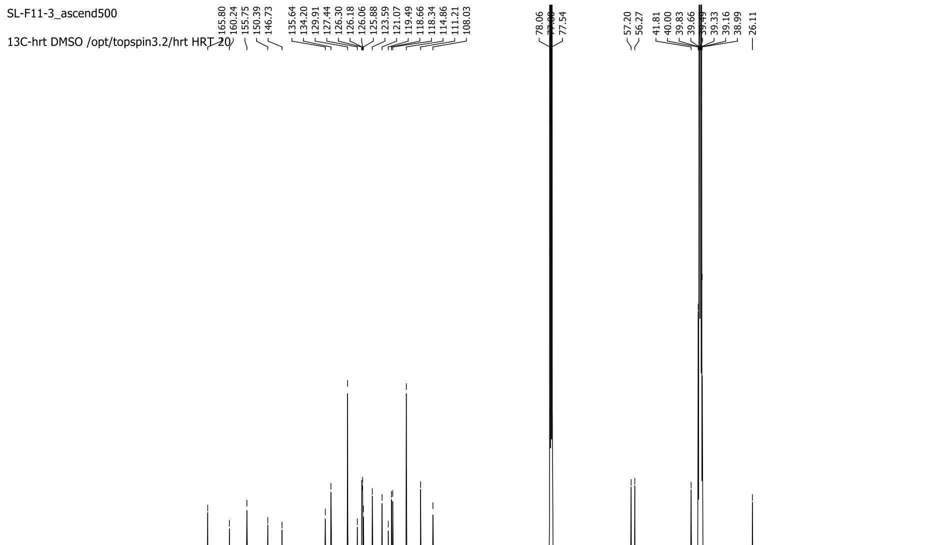

Figure S26: ^{13}C NMR spectrum of *(1S,4S)-4-((1H-indol-3-yl)methyl)-1-(4-hydroxybenzyl)-1,2-dihydro-6H-pyrazino[2,1-b]quinazoline-3,6(4H)-dione* **10** ($CDCl_3$, 75, MHz).

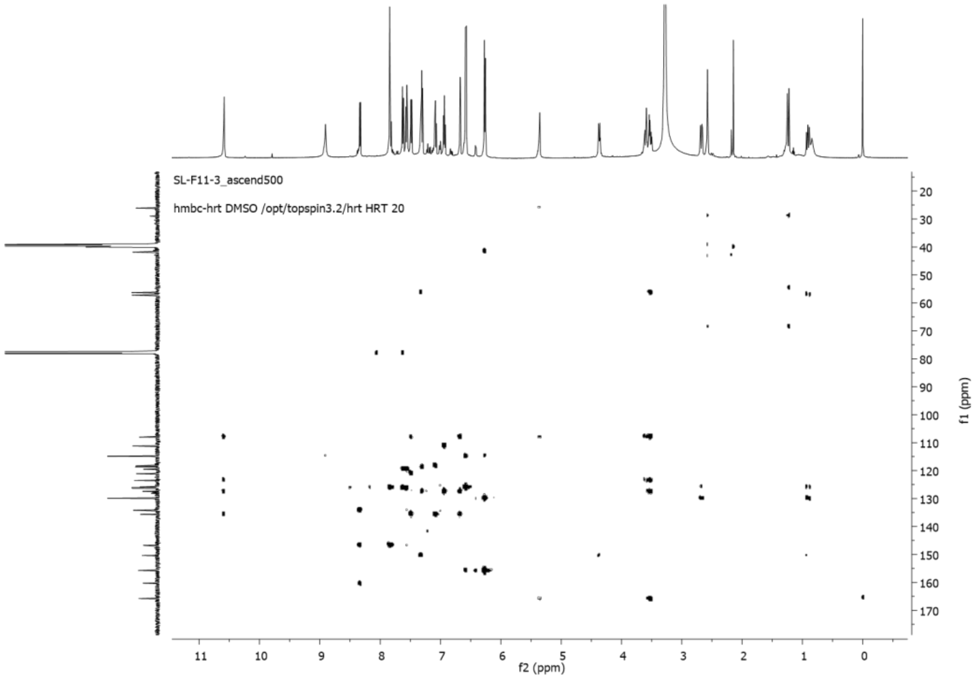

Figure S27: HMBCspectrum of *(1S,4S)-4-((1H-indol-3-yl)methyl)-1-(4-hydroxybenzyl)-1,2-dihydro-6H-pyrazino[2,1-b]quinazoline-3,6(4H)-dione* **10** ($CDCl_3$, 300, MHz).

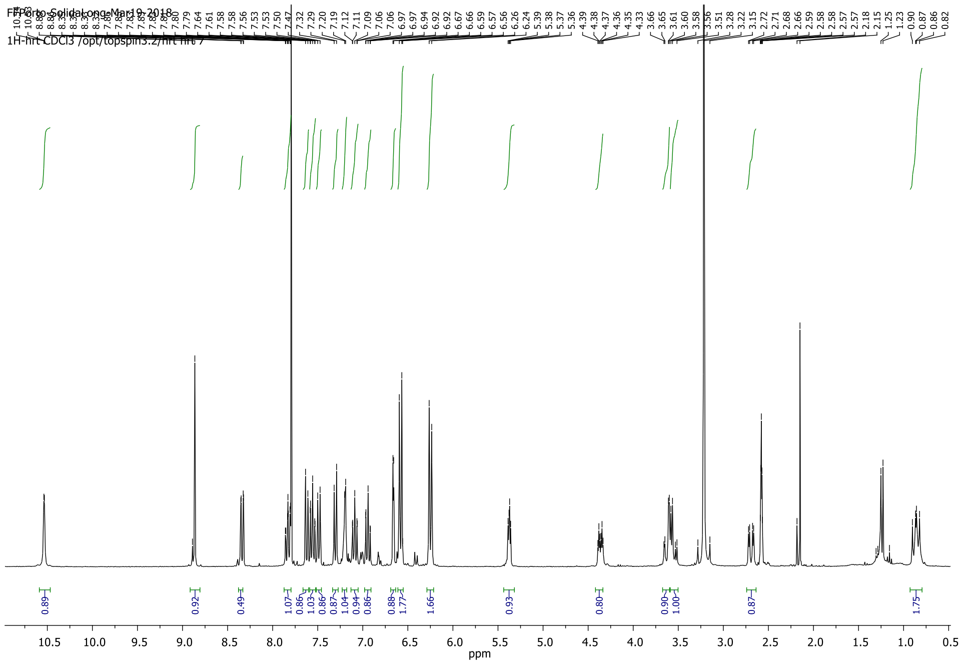

Figure S28: ^{1}H NMR spectrum of *(1R,4S)-4-((1H-indol-3-yl)methyl)-1-(4-hydroxybenzyl)-1,2-dihydro-6H-pyrazino[2,1-b]quinazoline-3,6(4H)-dione* (**11**) ($CDCl_3$, 300, MHz).

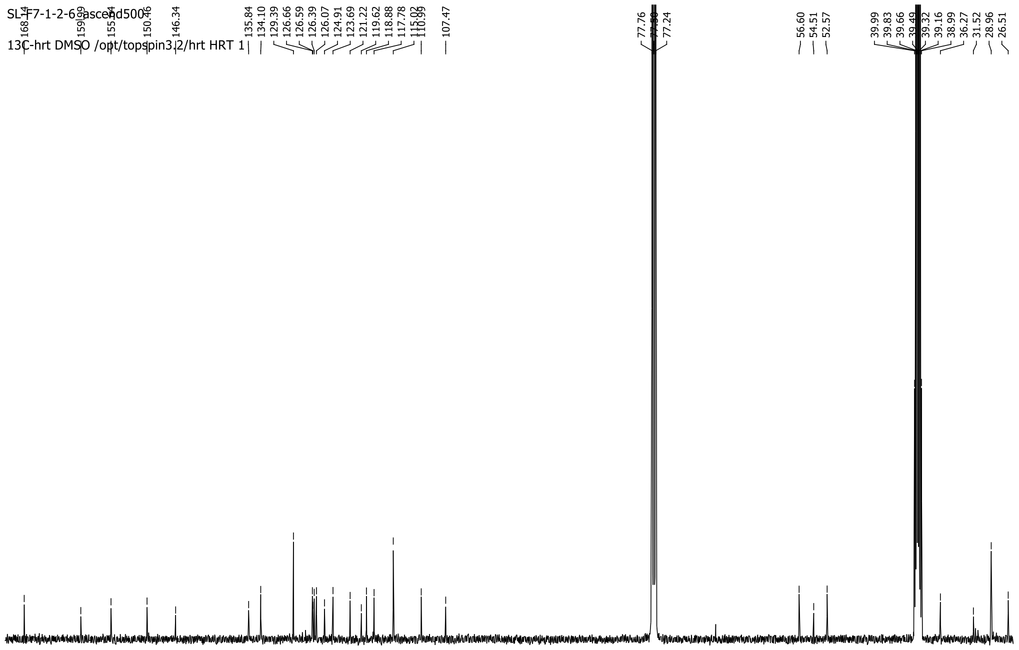

Figure S29: ^{13}C NMR spectrum of *(1R,4S)-4-((1H-indol-3-yl)methyl)-1-(4-hydroxybenzyl)-1,2-dihydro-6H-pyrazino[2,1-b]quinazoline-3,6(4H)-dione* (**11**) (CDCl3, 75, MHz).

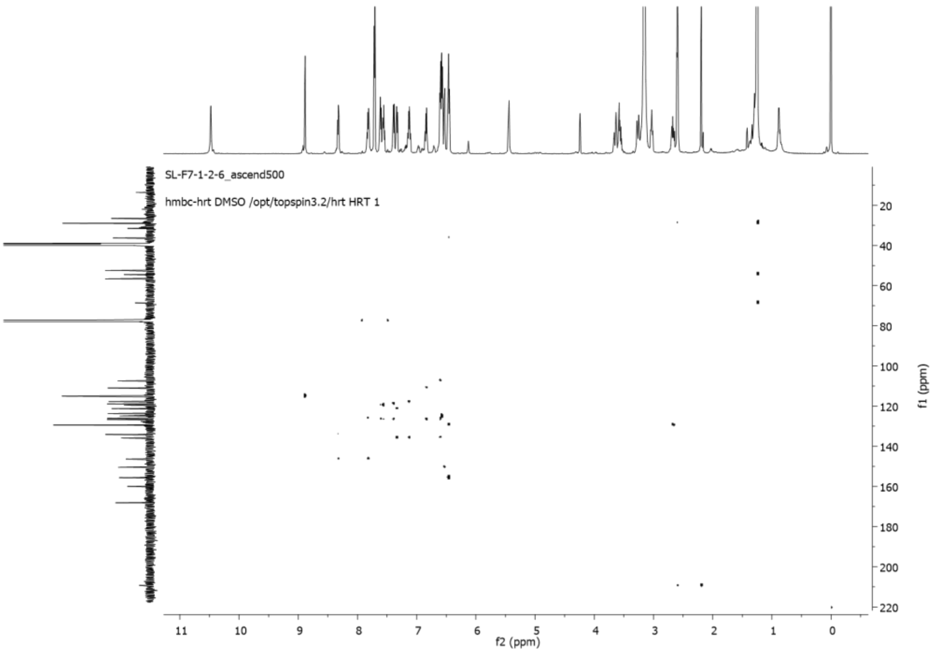

Figure S30: HMBC spectrum of *(1R,4S)-4-((1H-indol-3-yl)methyl)-1-(4-hydroxybenzyl)-1,2-dihydro-6H-pyrazino[2,1-b]quinazoline-3,6(4H)-dione* (**11**) (CDCl3, 300, MHz).

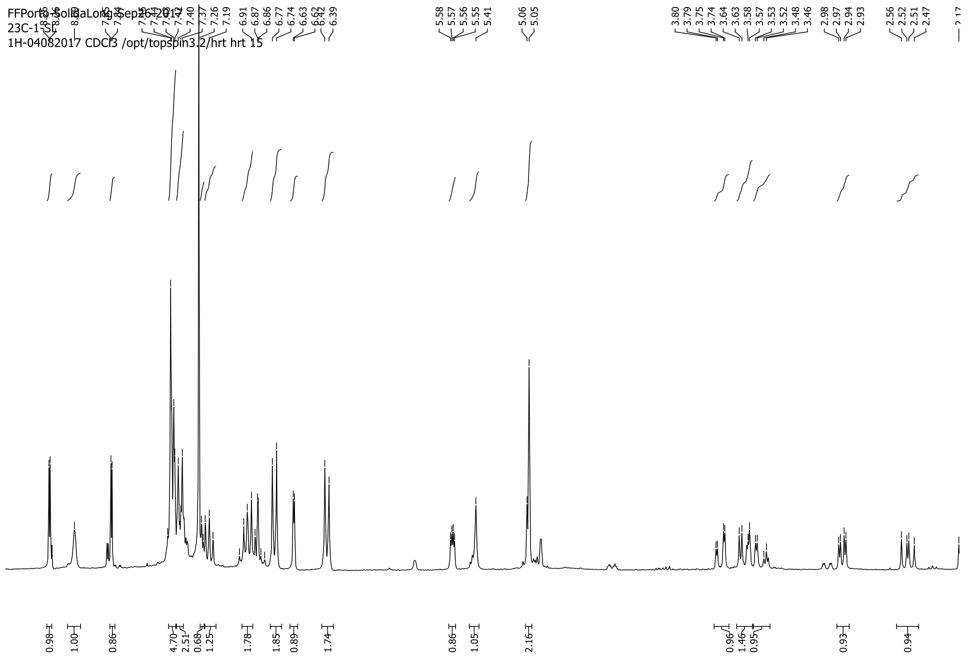

Figure S31: ^{1}H NMR spectrum of *(1S,4R)-4-((1H-indol-3-yl)methyl)-1-(4-(benzyloxy)benzyl)-8,10-dichloro-1,2-dihydro-6H-pyrazino[2,1-b]quinazoline-3,6(4H)-dione* (**12**) ($CDCl_3$, 300, MHz).

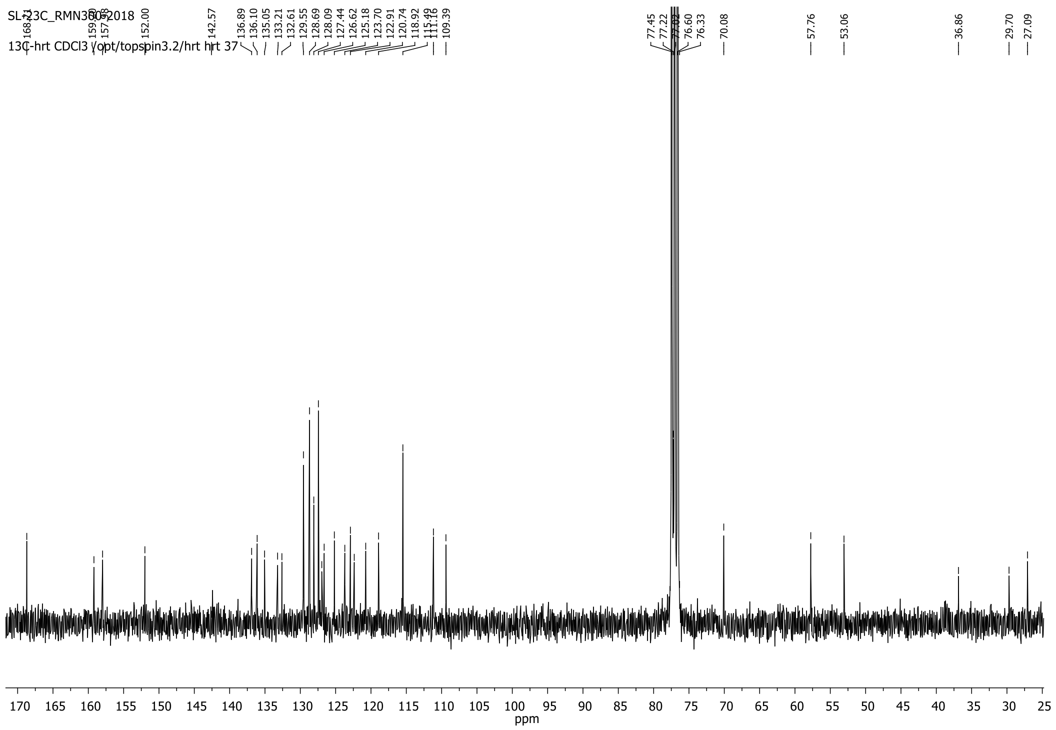

Figure S32: ^{13}C NMR spectrum of *(1S,4R)-4-((1H-indol-3-yl)methyl)-1-(4-(benzyloxy)benzyl)-8,10-dichloro-1,2-dihydro-6H-pyrazino[2,1-b]quinazoline-3,6(4H)-dione (***12**) ($CDCl_3$, 75, MHz).

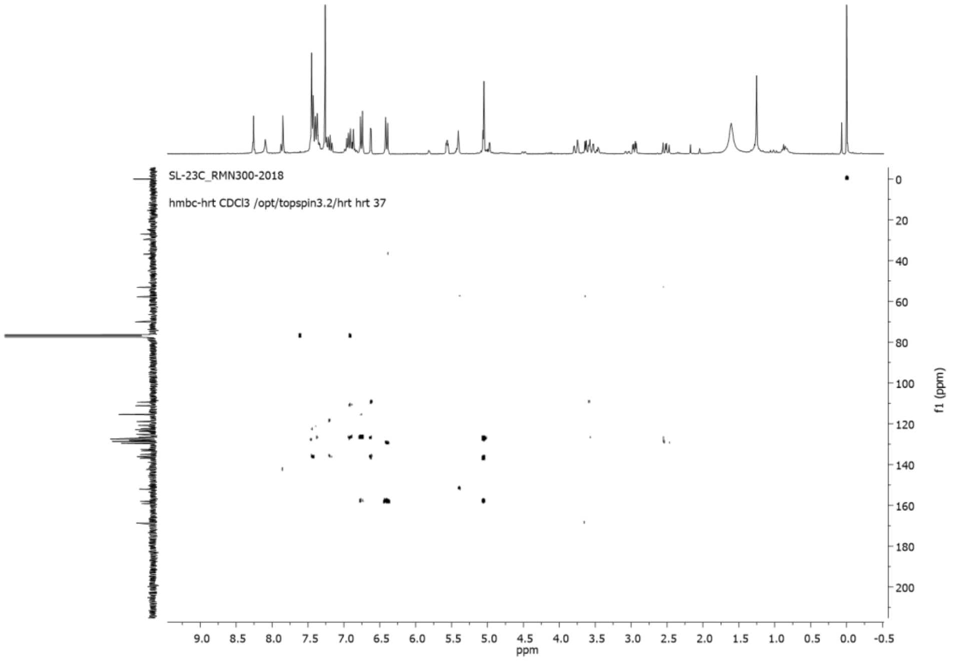

Figure S33: ^{13}C NMR spectrum of *(1S,4R)-4-((1H-indol-3-yl)methyl)-1-(4-(benzyloxy)benzyl)-8,10-dichloro-1,2-dihydro-6H-pyrazino[2,1-b]quinazoline-3,6(4H)-dione* (**12**) ($CDCl_3$, 300, MHz).

Mass Spectrum Molecular Formula Report

Means. m/z	#	Ion Formula	m/z	err [ppm]	Err [mDa]	rdb	N-rule
359,15053	M+H	C21H19N4O2	359.15025	0.779	0,28	14,5	even

Figure S34: HRMS of compound *(1R,4R)-4-((1H-indol-3-yl)methyl)-1-((R)-methyl)-1,2-dihydro-6H-pyrazino[2,1-b]quinazoline-3,6(4H)-dione* (**1**) (20, 300 V)

Mass Spectrum Molecular Formula Report

Meas. m/z	#	Formula	Score	m/z	err [mDa]	err [ppm]	mSigma	rdb	e⁻ Conf	N-Rule
399.18114	1	C 26 H 25 N O 3	51.48	399.18290	1.75	4.39	135.0	15.0	odd	ok
	2	C 24 H 23 N 4 O 2	100.00	399.18155	0.41	1.02	137.1	15.5	even	ok
401.19660	1	C 24 H 25 N 4 O 2	100.00	401.19720	0.60	1.50	5.8	14.5	even	ok
	2	C 26 H 27 N O 3	39.68	401.19855	1.94	4.84	10.7	14.0	odd	ok

Figure S35: HRMS of compound *(1S,4S)-4-((1H-indol-3-yl)methyl)-1-((S)-sec-butyl)-1,2-dihydro-6H-pyrazino[2,1-b]quinazoline-3,6(4H)-dione* (**2**) (20, 300 V)

Mass Spectrum Molecular Formula Report

Meas. m/z	#	Ion Formula	m/z	err [ppm]	err [mDa]	mSigma	Score	rdb	e^- Conf	N-Rule
401.19645	1	C24H25N4O2	401.19720	1.88	0.75	2.0	100.00	14.5	even	ok
423.17867	1	C24H24N4NaO2	423.17915	1.12	0.47	25.9	100.00	14.5	even	ok

Figure S36: HMRS of compound *(1R,4S)-4-((1H-indol-3-yl)methyl)-1-((S)-sec-butyl)-1,2-dihydro-6H-pyrazino[2,1-b]quinazoline-3,6(4H)-dione* (**4**) (20, 300 V)

Mass Spectrum Molecular Formula Report

Meas. m/z	#	Ion Formula	m/z	err [ppm]	err [mDa]	mSigma	Score	rdb	e^- Conf	N-Rule
401.19732	1	C24H25N4O2	401.19720	-0.29	-0.11	1.6	100.00	14.5	even	ok

Figure S37: HRMS of compound *(1S,4R)-4-((1H-indol-3-yl)methyl)-1-((S)-sec-butyl)-1,2-dihydro-6H-pyrazino[2,1-b]quinazoline-3,6(4H)-dione* (**5**) (20, 300 V)

Mass Spectrum Molecular Formula Report

Meas. m/z	#	Ion Formula	m/z	err [ppm]	err [mDa]	mSigma	Score	rdb	e^- Conf	N-Rule
419.15438	1	C23H23N4O2S	419.15362	-1.80	-0.76	4.3	100.00	14.5	even	ok
435.15042	1	C23H23N4O3S	435.14854	-4.33	-1.88	9.0	100.00	14.5	even	ok

FigureS38: HRMS of compound *(1R,4S)-4-((1H-indol-3-yl)methyl)-1-(2-(methylthio)ethyl)-1,2-dihydro-6H-pyrazino[2,1-b]quinazoline-3,6(4H)-diones* (**6**) (20, 300 V)

Mass Spectrum Molecular Formula Report

Meas. m/z	#	Ion Formula	m/z	err [ppm]	err [mDa]	mSigma	Score	rdb	e^- Conf	N-Rule
419.15257	1	C23H23N4O2S	419.15362	2.50	1.05	1.5	100.00	14.5	even	ok
441.13406	1	C23H22N4NaO2S	441.13557	3.43	1.51	n.a.	100.00	14.5	even	ok

Figure S39: HRMS of compound *(1R,4S)-4-((1H-indol-3-yl)methyl)-1-(2-(methylthio)ethyl)-1,2-dihydro-6H-pyrazino[2,1-b]quinazoline-3,6(4H)-diones* (**7**) (20, 300 V)

Mass Spectrum Molecular Formula Report

Meas. m/z	#	Ion Formula	m/z	err [ppm]	err [mDa]	mSigma	Score	rdb	e^- Conf	N-Rule
541.22211	1	C34H29N4O3	541.22342	2.41	1.30	9.5	100.00	22.5	even	ok

Figure S40: HRMS of compound *(1S,4S)-4-((1H-indol-3-yl)methyl)-1-(4-(benzyloxy)benzyl)-1,2-dihydro-6H-pyrazino[2,1-b]quinazoline-3,6(4H)-diones* (**8**) (20, 300 V)

Mass Spectrum Molecular Formula Report

Meas. m/z	#	Ion Formula	m/z	err [ppm]	err [mDa]	mSigma	Score	rdb	e⁻ Conf	N-Rule
541.22324	1	C34H29N4O3	541.22342	0.32	0.17	1.8	100.00	22.5	even	ok

Figure S41: HRMS of compound *(1R,4S)-4-((1H-indol-3-yl)methyl)-1-(4-(benzyloxy)benzyl)-1,2-dihydro-6H-pyrazino[2,1-b]quinazoline-3,6(4H)-diones* (**9**) (20, 300 V)

Mass Spectrum Molecular Formula Report

Meas. m/z	#	Ion Formula	m/z	err [ppm]	err [mDa]	mSigma	Score	rdb	e⁻ Conf	N-Rule
451.17711	1	C27H23N4O3	451.17647	-1.42	-0.64	20.0	100.00	18.5	even	ok
473.15639	1	C27H22N4NaO3	473.15841	4.28	2.02	n.a.	100.00	18.5	even	ok

Figure S42: HRMS of compound *(1S,4S)-4-((1H-indol-3-yl)methyl)-1-(4-hydroxybenzyl)-1,2-dihydro-6H-pyrazino[2,1-b]quinazoline-3,6(4H)-dione* (**10**) (20, 300 V)

Mass Spectrum Molecular Formula Report

Meas. m/z	#	Ion Formula	m/z	err [ppm]	err [mDa]	mSigma	Score	rdb	e⁻ Conf	N-Rule
451.17655	1	C27H23N4O3	451.17647	-0.18	-0.08	3.0	100.00	18.5	even	ok
473.15762	1	C27H22N4NaO3	473.15841	1.67	0.79	n.a.	100.00	18.5	even	ok

Figure S43: HRMS of compound *(1R,4S)-4-((1H-indol-3-yl)methyl)-1-(4-hydroxybenzyl)-1,2-dihydro-6H-pyrazino[2,1-b]quinazoline-3,6(4H)-dione* (11) (20, 300 V)

Mass Spectrum Molecular Formular Report

Means. m/z	#	Ion Formula	m/z	err [ppm]	Err [mDa]	rdb	N-rule
609,14269	M+H	C34H27Cl2N4O3	609.14547	-4.56	-2,78	22,5	even

Figure S44: HRMS of *(1S,4R)-4-((1H-indol-3-yl)methyl)-1-(4-(benzyloxy)benzyl)-8,10-dichloro-1,2-dihydro-6H-pyrazino[2,1-b]quinazoline-3,6(4H)-dione* (**12**) (20, 300 V)

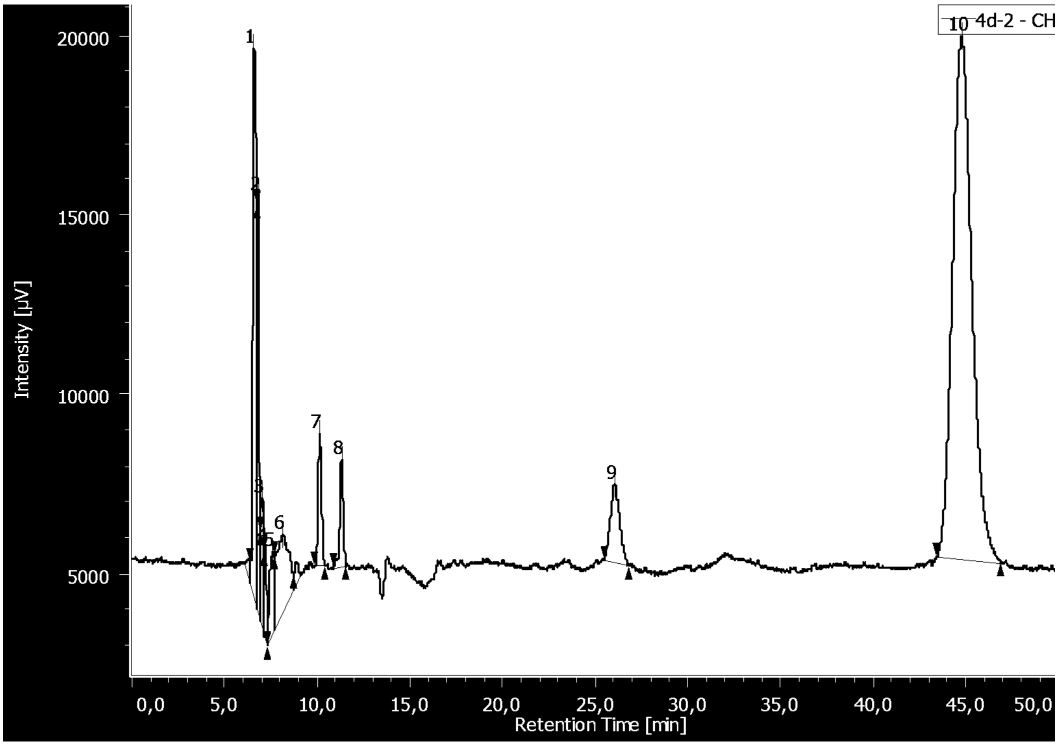

Figure S45: Chiral analysis of *(1R,4R)-4-((1H-indol-3-yl)methyl)-1-((R)-methyl)-1,2-dihydro-6H-pyrazino[2,1-b]quinazoline-3,6(4H)-dione* (**1**), Solvent: Hexan:MeOH,80:20; flowrate: 0.5 mL/min

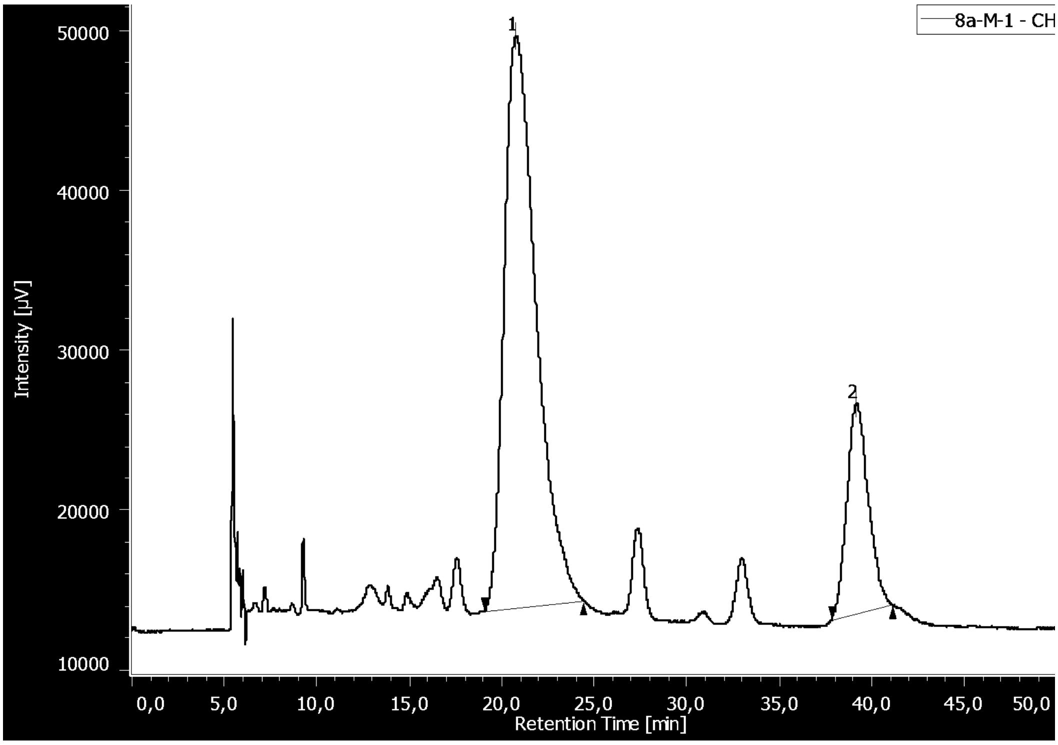

Figure S46: Chiral analysis of *(1S,4S)-4-((1H-indol-3-yl)methyl)-1-((S)-sec-butyl)-1,2-dihydro-6H-pyrazino[2,1-b]quinazoline-3,6(4H)-dione* (**2**), Solvent: Hexan:MeOH,80:20; flowrate: 0.5 mL/min

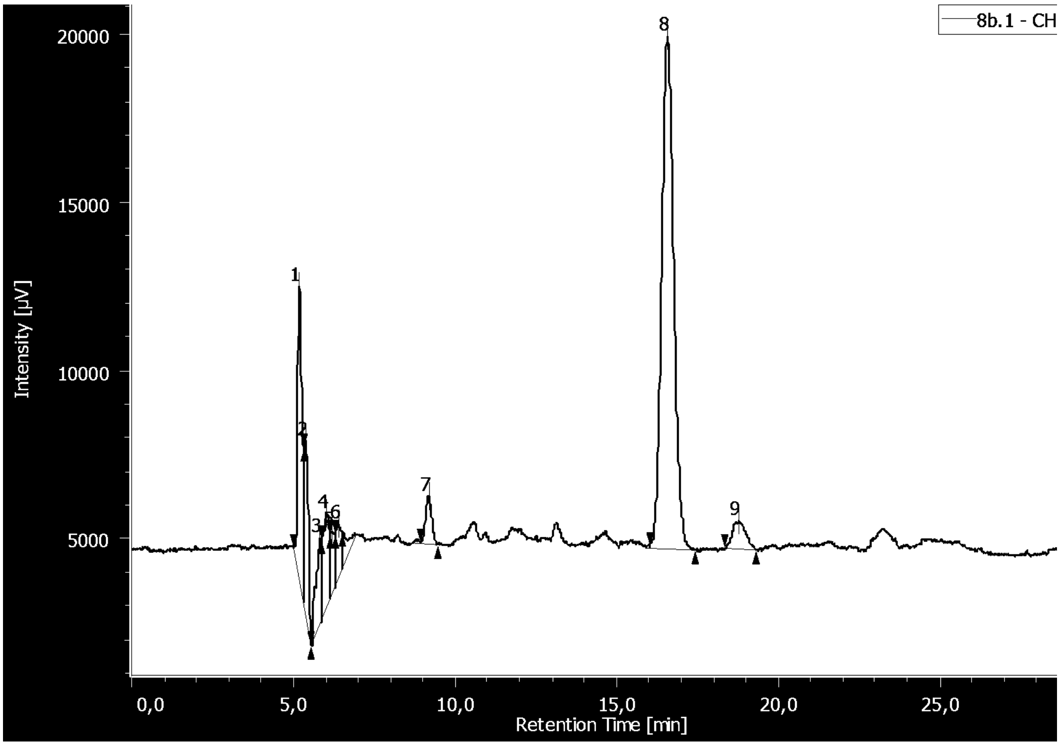

Figure S47: Chiral analysis of *(1R,4S)-4-((1H-indol-3-yl)methyl)-1-((S)-sec-butyl)-1,2-dihydro-6H-pyrazino[2,1-b]quinazoline-3,6(4H)-dione* (**4**), Solvent: Hexan:MeOH,80:20; flowrate: 0.5 mL/min

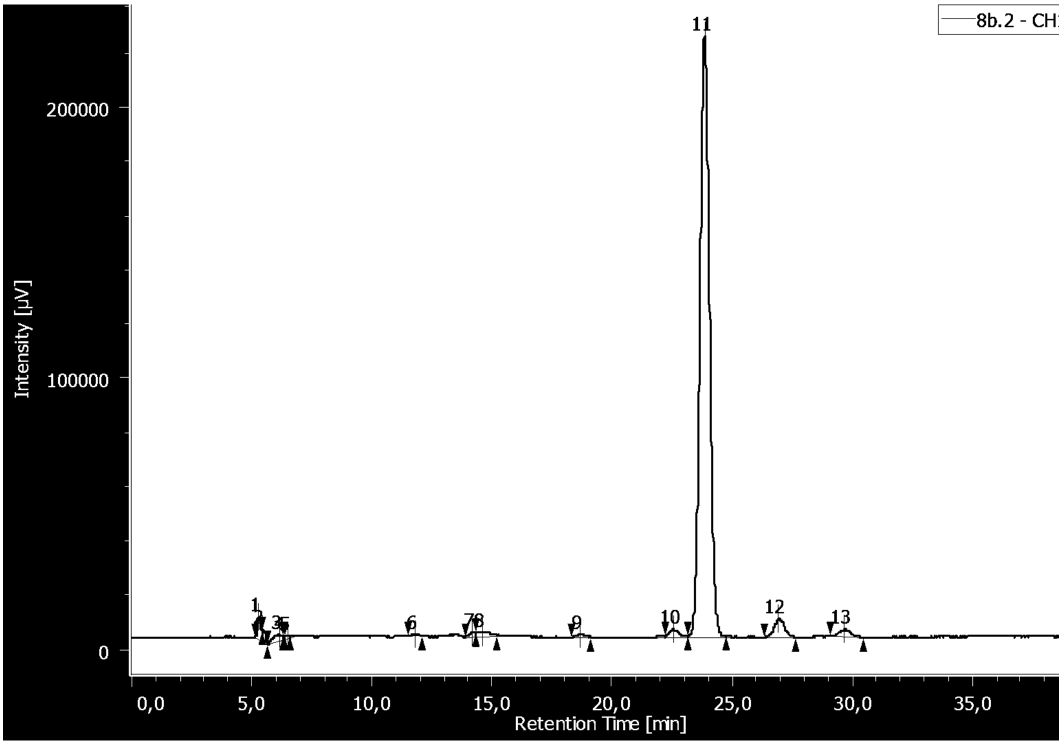

Figure S48: Chiral analysis of *(1S,4R)-4-((1H-indol-3-yl)methyl)-1-((S)-sec-butyl)-1,2-dihydro-6H-pyrazino[2,1-b]quinazoline-3,6(4H)-dione* (**5**), Solvent: Hexan:MeOH,80:20; flowrate: 0.5 mL/min

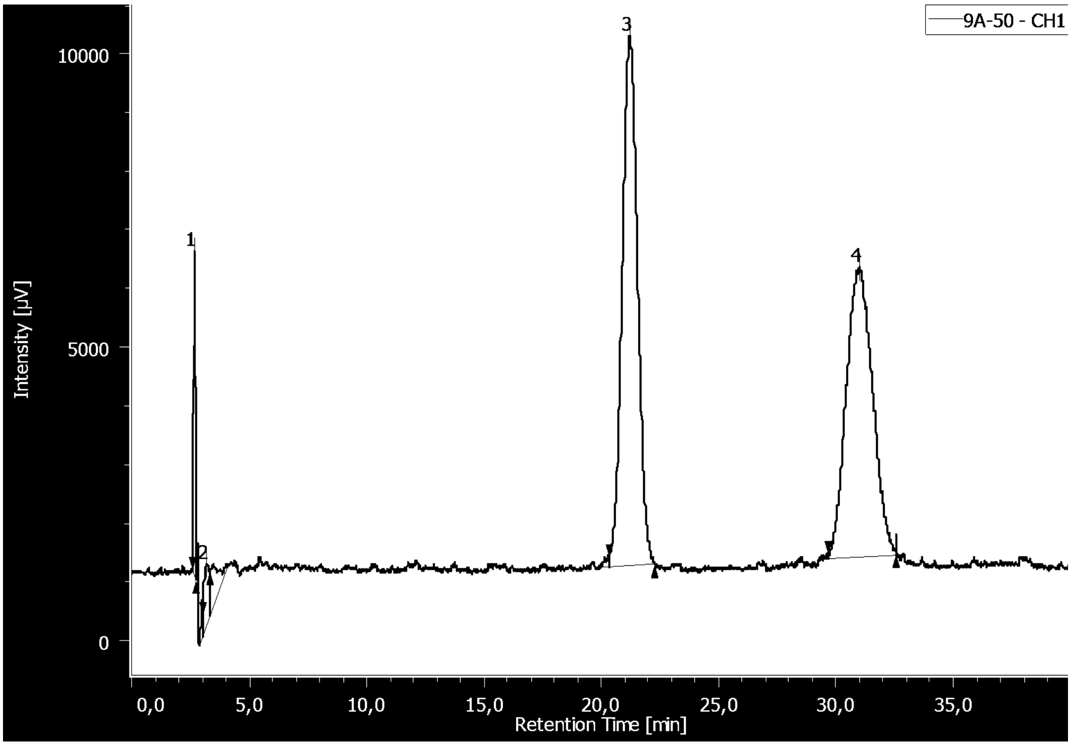

Figure S49: Chiral analysis of *(1R,4S)-4-((1H-indol-3-yl)methyl)-1-(2-(methylthio)ethyl)-1,2-dihydro-6H-pyrazino[2,1-b]quinazoline-3,6(4H)-diones* (**6**), Solvent: Hexan:MeOH,80:20; flowrate: 0.5 mL/min

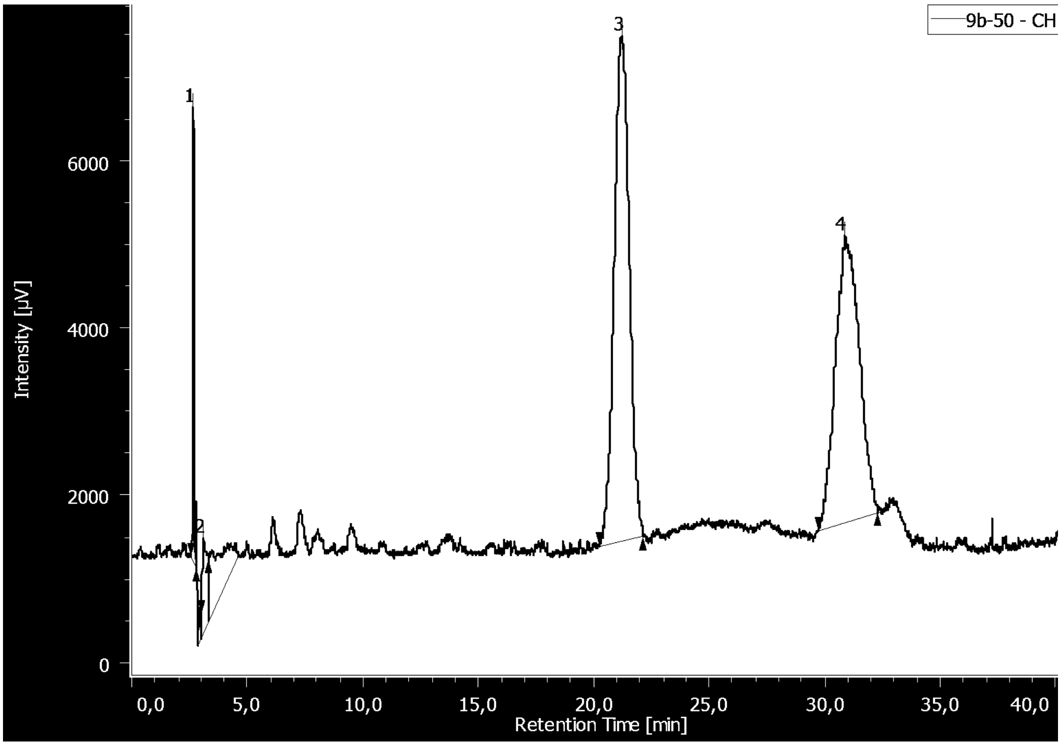

Figure S50: Chiral analysis of *(1R,4S)-4-((1H-indol-3-yl)methyl)-1-(2-(methylthio)ethyl)-1,2-dihydro-6H-pyrazino[2,1-b]quinazoline-3,6(4H)-diones* (**7**), Solvent: Hexan:MeOH,80:20; flowrate: 0.5 mL/min

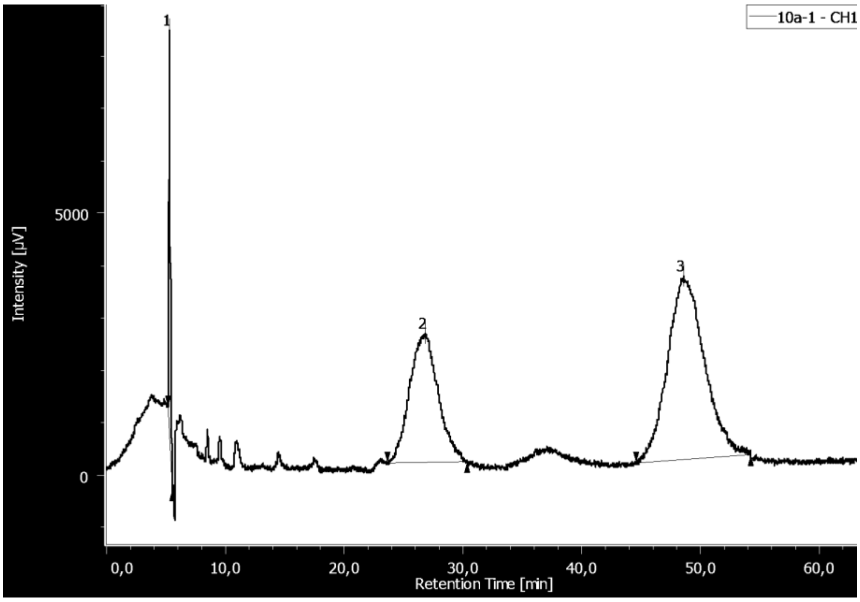

Figure S51: Chiral analysis of *(1S,4S)-4-((1H-indol-3-yl)methyl)-1-(4-(benzyloxy)benzyl)-1,2-dihydro-6H-pyrazino[2,1-b]quinazoline-3,6(4H)-diones* (**8**), Solvent: Hexan:MeOH,80:20; flowrate: 0.5 mL/min

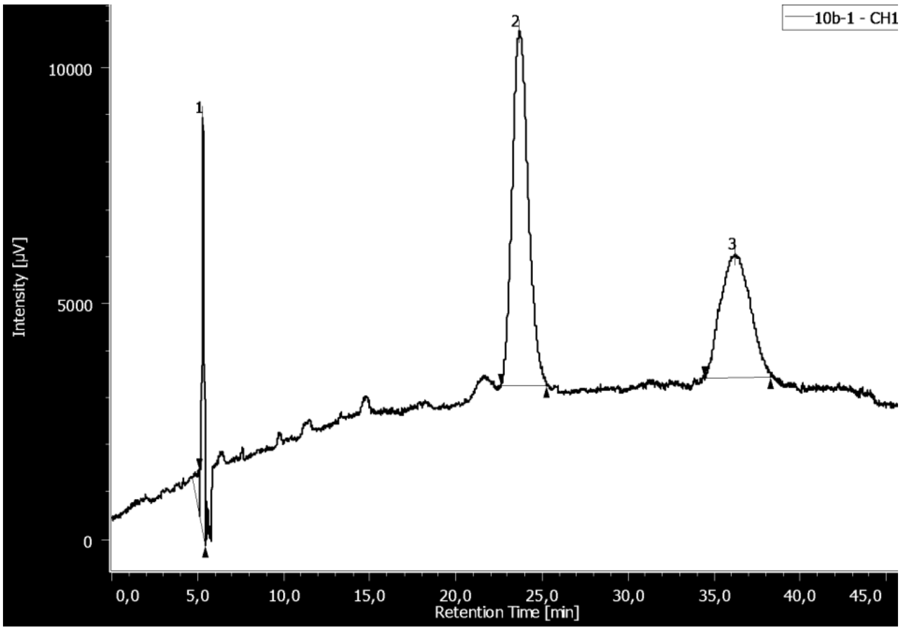

Figure S52: Chiral analysis of *(1R,4S)-4-((1H-indol-3-yl)methyl)-1-(4-(benzyloxy)benzyl)-1,2-dihydro-6H-pyrazino[2,1-b]quinazoline-3,6(4H)-diones* (**9**), Solvent: Hexan:MeOH,80:20; flowrate: 0.5 mL/min

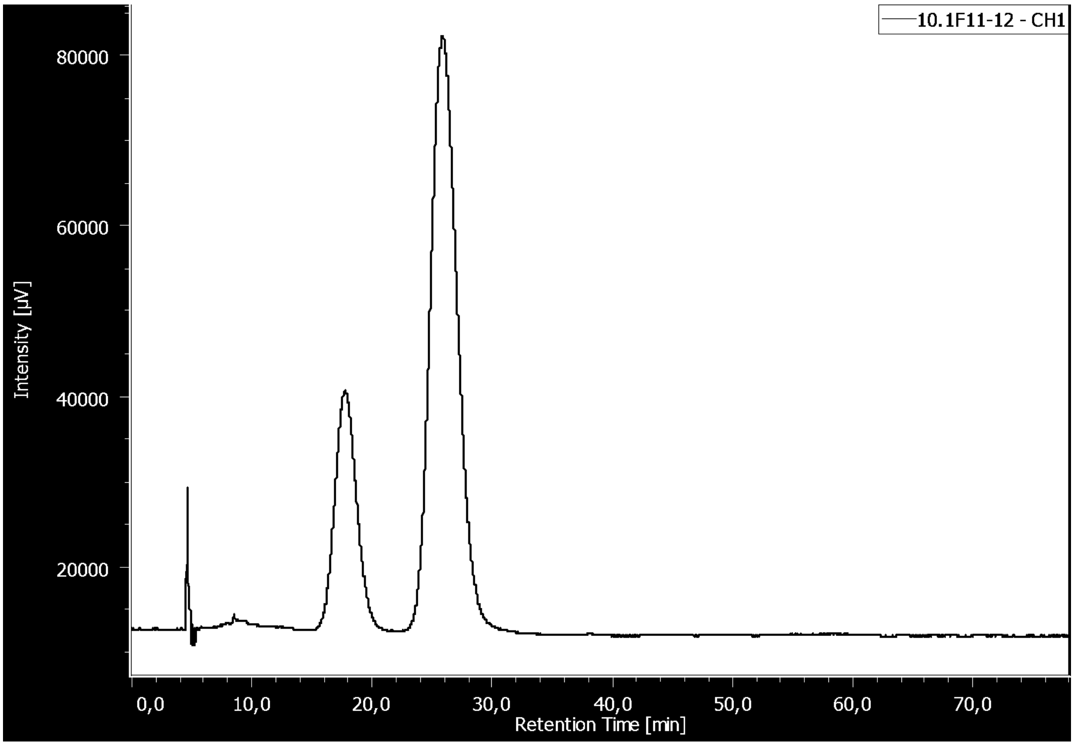

Figure S53: Chiral analysis of *(1S,4S)-4-((1H-indol-3-yl)methyl)-1-(4-hydroxybenzyl)-1,2-dihydro-6H-pyrazino[2,1-b]quinazoline-3,6(4H)-dione* (**10**), Solvent: Hexan:MeOH,80:20; flowrate: 0.5 mL/min

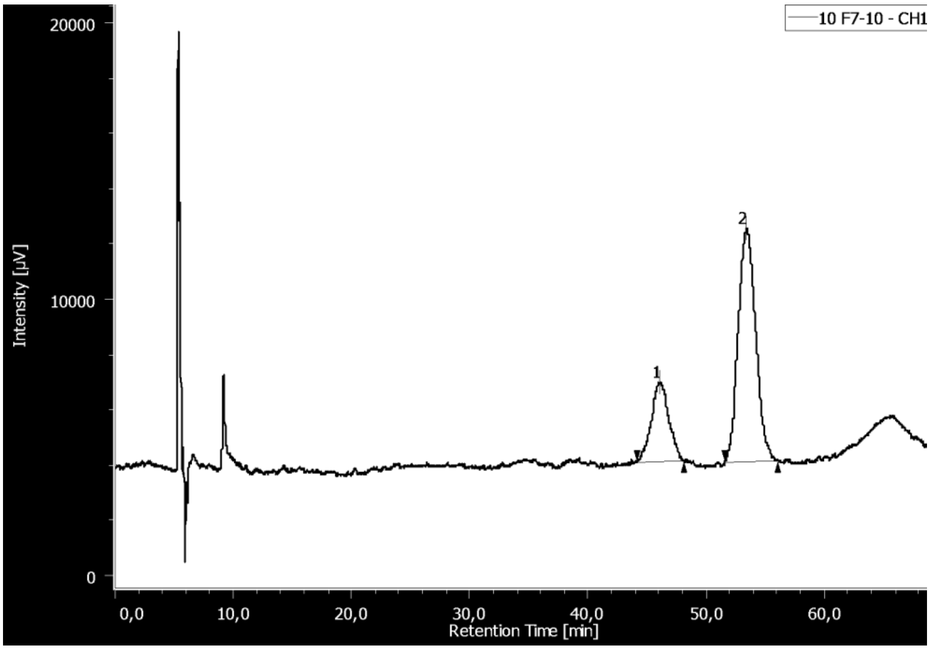

Figure S54: Chiral analysis of *(1R,4S)-4-((1H-indol-3-yl)methyl)-1-(4-hydroxybenzyl)-1,2-dihydro-6H-pyrazino[2,1-b]quinazoline-3,6(4H)-dione* (**11**), Solvent: Hexan:MeOH,80:20; flowrate: 0.5 mL/min

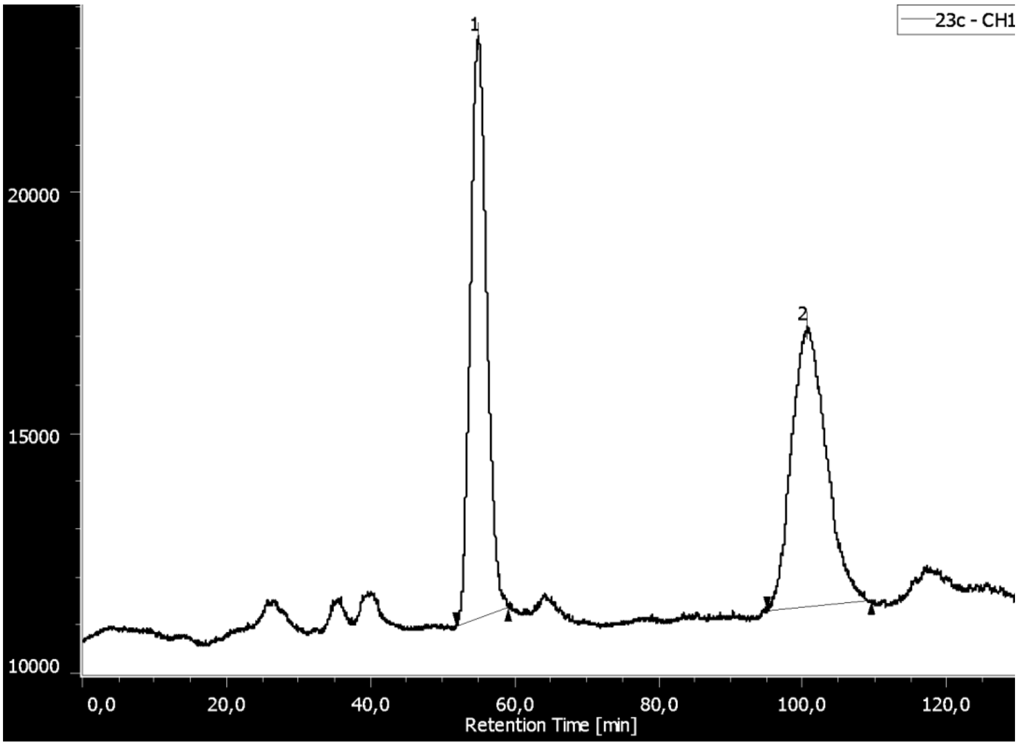

Figure S55: Chiral analysis of *(1S,4R)-4-((1H-indol-3-yl)methyl)-1-(4-(benzyloxy)benzyl)-8,10-dichloro-1,2-dihydro-6H-pyrazino[2,1-b]quinazoline-3,6(4H)-dione* (**12**), Solvent: Hexan:MeOH,80:20; flowrate: 0.5 mL/min

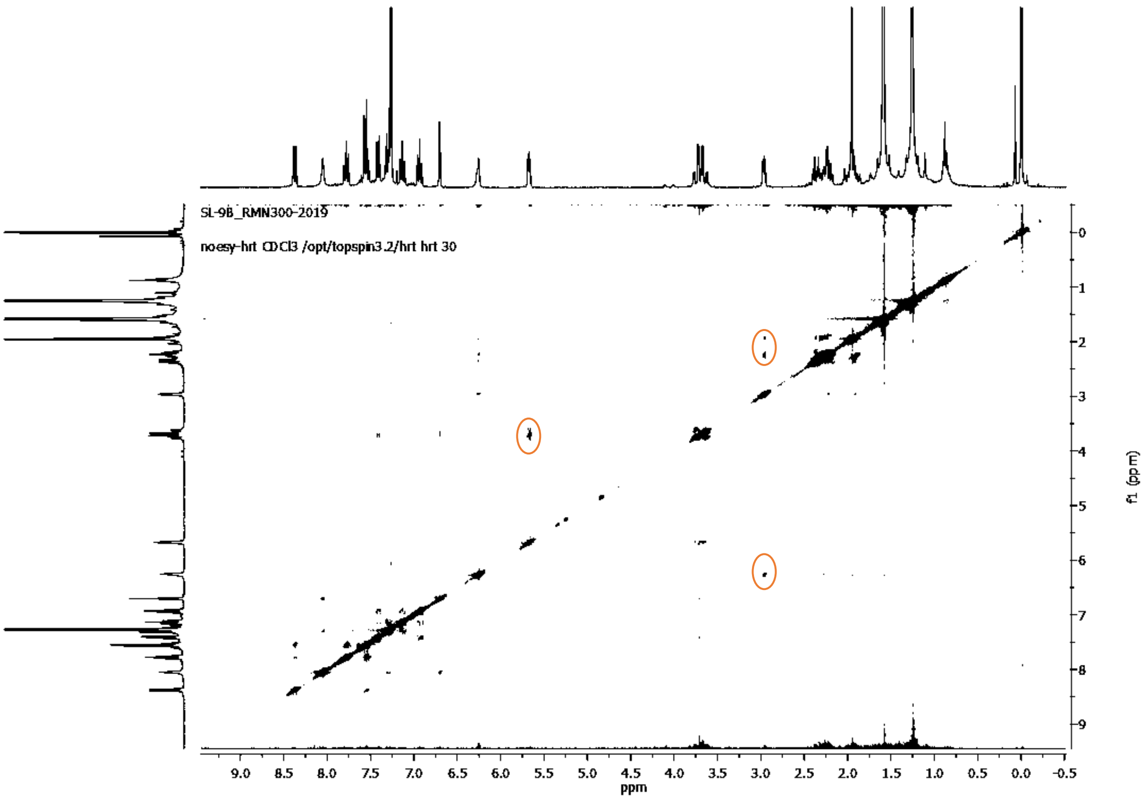

Figure S 56: The NOESY of compound *(1R,4S)-4-((1H-indol-3-yl)methyl)-1-(2-(methylthio)ethyl)-1,2-dihydro-6H-pyrazino[2,1-b]quinazoline-3,6(4H)-diones* (**6**) ($CDCl_3$, 300, MHz)..

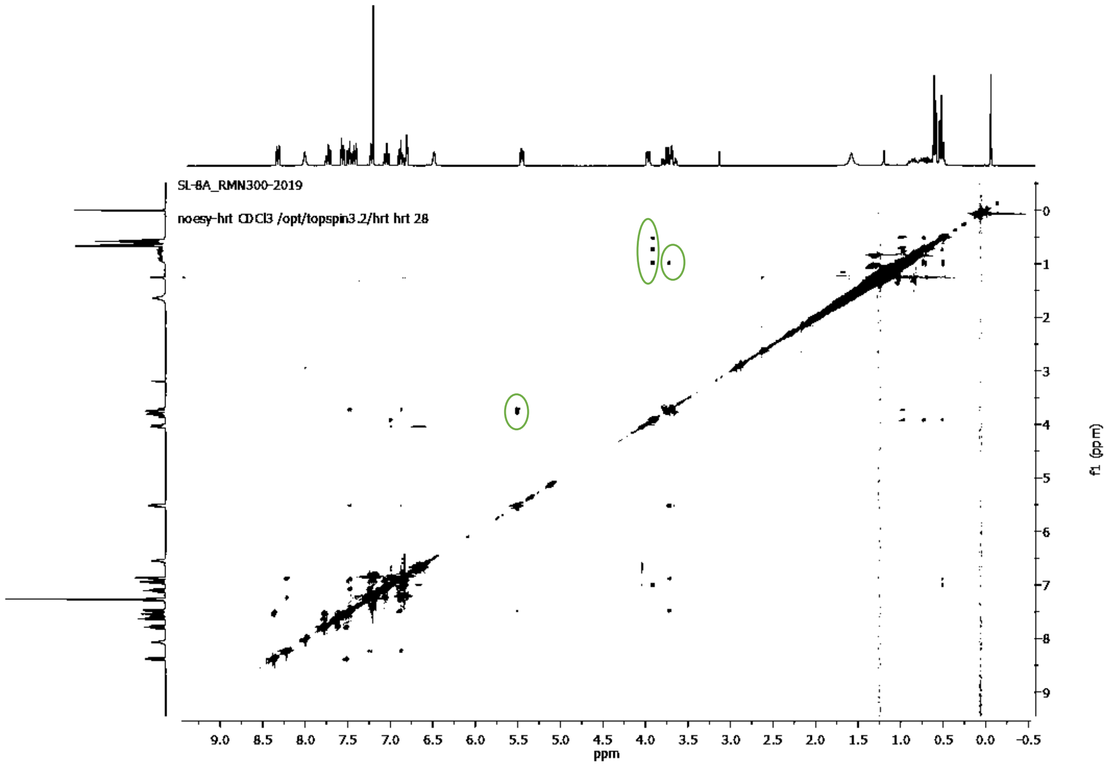

Figure S 57: The NOESY *(1S,4S)-4-((1H-indol-3-yl)methyl)-1-((S)-sec-butyl)-1,2-dihydro-6H-pyrazino[2,1-b]quinazoline-3,6(4H)-dione* (**2**) ($CDCl_3$, 300, MHz).
